# CryoEM reveals the complex self-assembly of a chemically driven disulfide hydrogel†

**DOI:** 10.1039/d3sc05790a

**Published:** 2023-12-18

**Authors:** Paul Joshua Hurst, Justin T. Mulvey, Rebecca A. Bone, Serxho Selmani, Redford F. Hudson, Zhibin Guan, Jason R. Green, Joseph P. Patterson

**Affiliations:** a Department of Chemistry, University of California, Irvine Irvine California 92697 USA patters3@uci.edu; b Center for Complex and Active Materials, University of California, Irvine Irvine California 92697 USA; c Department of Materials Science and Engineering, University of California, Irvine Irvine California 92697 USA; d Department of Chemistry, University of Massachusetts Boston Boston Massachusetts 02125 USA; e Department of Computer Science, University of California, Irvine Irvine California 92697 USA; f Department of Chemical and Biomolecular Engineering, University of California, Irvine Irvine California 92697 USA; g Department of Biomedical Engineering, University of California, Irvine Irvine California 92697 USA; h Department of Physics, University of Massachusetts Boston Boston Massachusetts 02125 USA

## Abstract

Inspired by the adaptability of biological materials, a variety of synthetic, chemically driven self-assembly processes have been developed that result in the transient formation of supramolecular structures. These structures form through two simultaneous reactions, forward and backward, which generate and consume a molecule that undergoes self-assembly. The dynamics of these assembly processes have been shown to differ from conventional thermodynamically stable molecular assemblies. However, the evolution of nanoscale morphologies in chemically driven self-assembly and how they compare to conventional assemblies has not been resolved. Here, we use a chemically driven redox system to separately carry out the forward and backward reactions. We analyze the forward and backward reactions both sequentially and synchronously with time-resolved cryogenic transmission electron microscopy (cryoEM). Quantitative image analysis shows that the synchronous process is more complex and heterogeneous than the sequential process. Our key finding is that a thermodynamically unstable stacked nanorod phase, briefly observed in the backward reaction, is sustained for ∼6 hours in the synchronous process. Kinetic Monte Carlo modeling show that the synchronous process is driven by multiple cycles of assembly and disassembly. The collective data suggest that chemically driven self-assembly can create sustained morphologies not seen in thermodynamically stable assemblies by kinetically stabilizing transient intermediates. This finding provides plausible design principles to develop and optimize supramolecular materials with novel properties.

## Abstract 2

We elucidate the mechanisms of chemically driven self-assembly processes, demonstrating how synchronous assembly–disassembly reactions can stabilize transient structures and create morphologies that differ from conventional assemblies.

## Graphical abstract

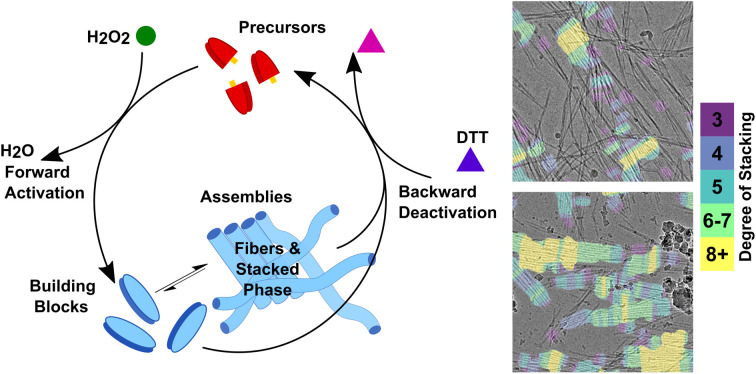

## Introduction

Living organisms produce materials which are highly adaptive to their environment.^1–5^ A key feature of these systems is that they do not operate in deep thermodynamic wells where a significant amount of energy would be required to change their structure.^5–8^ Inspired by this feature of biology, synthetic chemists have sought to create molecular systems that can begin to mimic this adaptive behavior. One widely studied approach, which is sometimes referred to as “dissipative self-assembly”,^9^ is to create a chemically driven self-assembly process in which two simultaneous reactions take place, a forward and backward reaction. The forward reaction converts molecule A, which does not undergo self-assembly, into molecule B, which does undergo self-assembly. The backward reaction converts molecule B into molecule A.^5^ These chemically driven processes have been developed using small molecules, light or electricity to control the reactions.^6,10–26^ Microtubules, which are part of the cytoskeleton of eukaryotic cells, are often cited as an inspiration for the development of these systems. This analogy is in large part due to the fact that GTP drives the polymerization of microtubules, while the hydrolysis of GTP to GDP induces their depolymerization.^2,5,27,28^ Despite the great interest in the development of these synthetic chemically driven systems, it has been noted that the complexity of the biological materials that inspired this work is far greater than these synthetic systems,^9^ to the point where it is currently unclear if they are substantially different from conventional assemblies.^29^ This lack of clarity is largely due to our inability to readily provide detailed mechanistic information on the chemically driven self-assembly processes. Consequently, there is a need to compare chemically driven and conventional assemblies to provide guidance on their continued development. One potentially productive approach would be to study the forward and reverse reaction sequentially (A to B followed by B to A) and compare this to the synchronous process (A and B can simultaneously interconvert). However, this comparison is often challenging due to the difficulty in decoupling the forward and backward reaction. In most cases, one or more of the steps is dependent on the solvent, or both the forward and backward reaction are driven by the same reagent.^10,11^ For example, Rizzuto *et al.*^30^ developed a supramolecular DNA fiber system in which annealing by slow proton dissipation selects for morphologies otherwise inaccessible by conventional self-assembly. Comparison of the forward reaction to the synchronous reactions was key to showing that a synchronous process can lead to the formation of higher order structures. However, with this system, it was not possible to isolate the backward reaction from the synchronous process as the forward assembly reaction cannot be “turned off”.

Recently, Ogden and Guan developed a hydrogelator chemically driven by redox reactions that uses separate small molecule reagents for the forward and backward reactions, making it possible to isolate them.^23^ In the forward reaction, an aryl-containing cysteine-based thiol (CSH) is oxidized by H_2_O_2_ into its disulfide dimer (CSSC), which spontaneously self-assembles into fibers forming a hydrogel. Simultaneously, at an initially slower rate, excess dithiothreitol (DTT) reduces the CSSC back to CSH disassembling the fibers. As the H_2_O_2_ depletes, the system returns to CSH. Here, we directly compare the sequential (forward followed by backward) and synchronous assembly processes for this disulfide-based hydrogelator with time-resolved cryoEM.^31^ This comparison reveals that the synchronous process is substantially more complex than the sequential process. Furthermore, we show that thermodynamically unstable, yet highly ordered, structures in the sequential process can be stabilized for long periods (hours) by the synchronous reactions. To analyze this behavior, we developed an image analysis algorithm to quantify the evolution of the highly ordered phase. We also implemented and optimized a kinetic model for stochastic simulations of the transient assembly to establish the importance of physical kinetics and reaction cycles in creating and maintaining the unstable phase.

## Results

The synchronous CSH/CSSC process is maintained by the presence of both H_2_O_2_ (oxidant) and DTT (reductant). Thus, through the inclusion or exclusion of H_2_O_2_ and DTT, we can isolate and study the forward (F) oxidation of CSH, the backward (B) reduction of CSSC, and the synchronous (S) system separately (Table 1) with the goal of comparing the sequential (F followed by B) and transient chemically driven processes. Note: here we define our system as transient because the hydrogel only persists for a finite period.^23^ For each process, multiple time points were sampled for cryoEM (see Table 1 and experimental information for more details). Time-resolved ultra-performance liquid chromatography (UPLC) and rheology experiments were performed to determine the conversion times and obtain bulk data on gel formation and strength (Fig. S1–S5†). All experiments were performed at a pH of 6.0 in a buffer solution.

**Table 1:** Experimental parameters for the forward (assembly) and backward (disassembly) processes as well as the synchronous (transient self-assembly) process for CSH/CSSC system. All experiments were conducted at a pH of 6.0. B is performed from a completed sample of F and the collection time builds off F

Run^a^	Initial materials	#CryoEM time points	Collection time range
F	10 mM CSH, 10 mM H_2_O_2_	4	8–1500 s
B	5 mM CSSC (F after 1500 s), 200 mM DTT	4	1575–3375 s (& 21 days)
S	10 mM CSH, 150 mM H_2_O_2_, 200 mM DTT	22	8–24 000 s (& 19 days)

a F = forward, B = backward, S = synchronous.

**Fig. 1:**
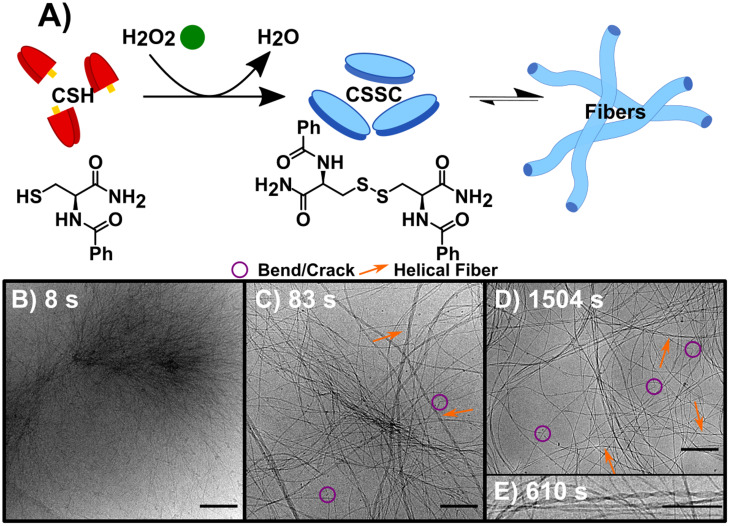
The forward reaction (F) converting CSH to assembling CSSC. (A) Reaction scheme, using H_2_O_2_ to oxidize the thiol CSH (red) to the disulfide CSSC (blue) leading to fiber formation. (B) CryoEM image 8 s into the assembly of CSSC shows the presence of centrosome-like structures. (C) CryoEM image at 83 s showing fibers become more well defined with a variety of diameters and twists and helical structures. (D) CryoEM image at 1504 s, fibers are more evenly spaced and remain as hydrogels in thermodynamic equilibrium. Legend provided: purple circles showing examples of bends and cracks in fibers and orange arrows to highlight helical fibers. (E) Inset of cryoEM image at 610 s of a helical fiber, a structure that is present after the initial time point. Scale bars are 200 nm except for (E), which is 10 nm.

In the sequential process, the forward reaction rapidly forms fibers. This reaction, F (Fig. 1A), was studied by the oxidation of CSH (10 mM) using H_2_O_2_ (10 mM). The gel storage modulus quickly increases until remaining constant after 1500 s indicating maximum conversion (Fig. S1†). CryoEM images of F and control experiments reveal the presence of long flexible nanofibers (see experimental controls in ESI†). The images show that the nanofibers (diameter ≈ 5 nm) can coil to form thicker hierarchical fibers (Fig. 1B–E, S6 and S7†). As the average molecular length of CSSC is approximately 14 Å (Fig. S8†), the base fiber diameter is about three to four times thicker than the length of a CSSC molecule. This indicates that the fibers are formed from multiple layers of CSSC molecules. Time-resolved cryoEM of F reveals that the fibers initially form from high density regions (*t* = 8 s, Fig. 1B and S9†). From 83 s to 1504 s the fibers become more uniformly distributed, likely driven by translational entropy (Fig. 1C and D). There stable samples showed a minor population (<10%) of these fibers have a helical structure with a pitch of 5.7 ± 0.7 nm (*n* = 218, see Fig. 1E). Although this population was minor, 96% of the images had at least one helical fiber present. Some fibers display bends and cracks which may indicate rearrangement occurs even after the hydrogel is stable (Fig. 1C and D).

**Fig. 2:**
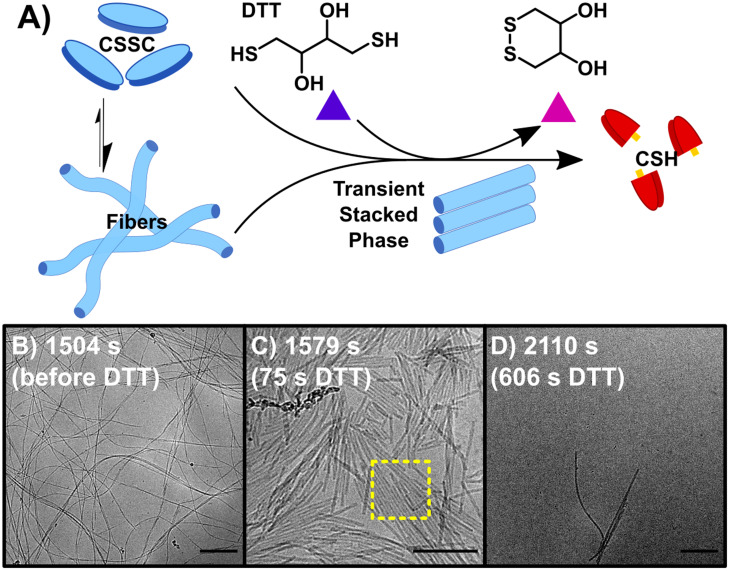
The backward reaction (B) of CSSC back to the CSH precursor. (A) The addition of DTT causes the breakdown of CSSC fibers back into CSH with the appearance of transient stacked nanorods. (B) CryoEM image of fibers before the addition of DTT. (C) CryoEM image at 1579 s, 75 s after the addition of DTT, fibers break up into nanorods with some of these rods showing 2-D stacking. (D) CryoEM image at 2110 s, most fibers have disassembled as the solutions return to a sol state, however a few fibers/nanorods can be seen in samples by cryoEM indicating that a small number of fibers could be kinetically trapped. Scale bars are 200 nm.

Initiating the backward reaction causes the quick disassembly of any fibers formed and transiently creates a thermodynamically unstable phase. The backward reaction (B) was carried out by taking a stable sample of the sample F (10 mM CSH, 10 mM H_2_O_2_) at 1500 s, consisting of CSSC, and treating it with 200 mM DTT (Fig. 2A). UPLC shows >99% conversion from CSSC to CSH was reached in 1200 s and rheological data shows a plateau in the storage modulus around 1200–1800 s (Fig. S2†). Time resolved cryoEM shows the breakdown of fibers (aspect ratio ≫ 25) into nanorods (aspect ratio 3–25), Fig. 2B–D.^32^ At 1575 s (75 s after addition of DTT), the presence of a stacked nanorod phase is also observed (Fig. 2C). The stacks are also observed in a control where 5 mM of H_2_O_2_ was used to ensure full reaction of the H_2_O_2_ before DTT addition (Fig. S10†). Importantly, these stacks are not observed in any subsequent time points, indicating this phase is thermodynamically unstable. After the stacked nanorod phase breaks down (*t* > 75 s after addition of DTT) only minor clustering of 2–3 nanorods/fibers is seen and the majority of the cryoEM grid area is void of fibers (Fig. 2D). CryoEM data show that trace fibers persist 21 days after initiation indicating that either a small number of fibers are kinetically trapped, an observation which has been made previously in other systems,^33^ or that there is a small amount of air oxidation. With the observation of a short-lived unstable phase, a logical question arises: can the lifetime of this phase be prolonged by the forward and backward reactions occurring simultaneously?

**Fig. 3:**
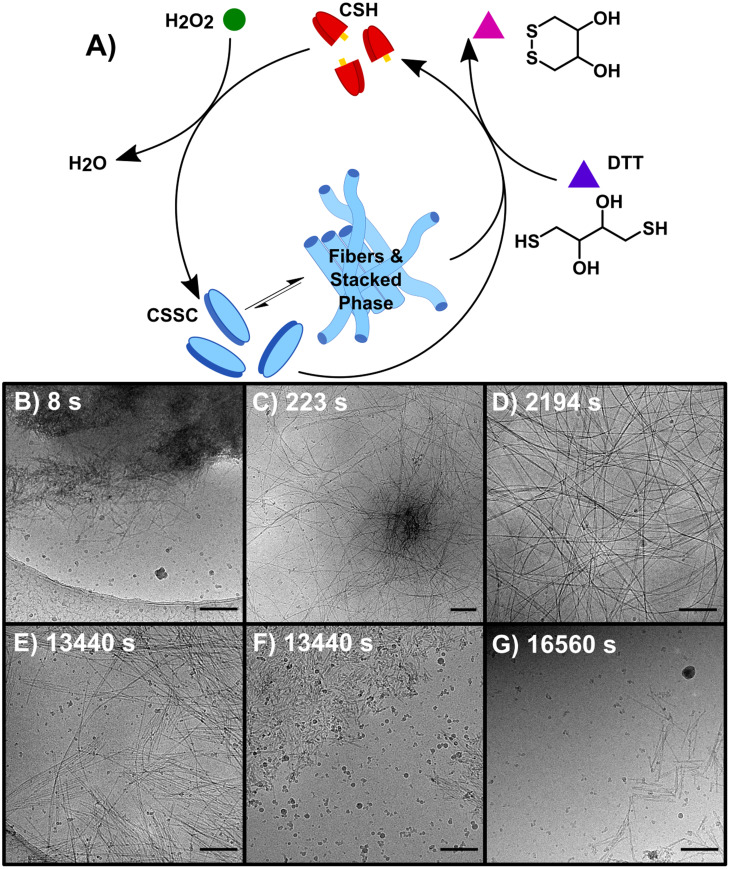
The synchronous system (S) which allows for the interconversion between non-assembling CSH and CSSC. (A) The reaction is sustained by the oxidation of CSH to CSSC by H_2_O_2_ and the simultaneous reduction of CSSC assembling CSH by DTT. (B–G) CryoEM images of representative structures of D (B) 8 s (C) 223 s (D) 2194 s (E and F) 13 440 s (G) 16 560 s. Scale bars are 200 nm.

Performing the forward and backward reactions synchronously (S) leads to a more complex process and the stabilization of the thermodynamically unstable phase. S was initiated by adding 150 mM H_2_O_2_ to a solution of 10 mM CSH and 200 mM DTT (Fig. 3A). H_2_O_2_ reacts with both CSH and DTT, however the rate constant for the reaction with CSH is four orders of magnitude larger than the rate constant for the reaction with DTT (Table S2†). S was studied over 6+ hours, a period based on previous kinetic data.^23^ A *t* = 0 sample prior to the addition of H_2_O_2_ showed no precursor structures (Fig. S11†). At 8 s, cryoEM shows the formation of unstructured aggregates with nanorod-like structures at the surface (Fig. 3B and S12†) similar to observations in the early stages of F (Fig. S9†). Although most fibers appear to form from these nucleation hot spots, individual isolated fibers were also observed (Fig. S12†), indicating that some fibers may form from a unimer growth mechanism. At 44 s, high density fiber regions were observed, which we interpret to have emerged from the unstructured aggregates (Fig. 3C). As time progresses (*t* > 225 s), these high-density fiber regions become less common as the fibers spread out (Fig. 3D). This observation is supported by the increase in gel strength as the reaction proceeds, indicating the development of a hydrogel network (Fig. S3†). The synchronous system forms long nanofibers that coil, comparable to F, including the presence of helical fibers (pitch = 5.1 ± 0.7 nm, *n* = 231). At *t* > 460 s, samples contain a stacked fibers/rod phase comparable to the stacked phase observed in B. The stacked phase is observed from 460 s to 23 400 s (6.5 h) (Fig. S13†), which shows that the synchronous chemistry can stabilize a thermodynamically unstable phase for ∼6 hours.

Compared to the sequential process, F and B, our observations make clear that the synchronous process, S, produces a system that is more heterogeneous and complex. For instance, following the emergence of the stacked phase (*t* > 460 s), many examples of long flexible dispersed nanofibers are observed that are morphologically similar to those found in F. As disassembly dominates, (*t* > 4300 s) the stacked phase becomes more prevalent (Fig. 3E) as well as a phase of disordered nanorod-aggregates (Fig. 3F). At later times, the prevalence of fibers and rods becomes sparse (Fig. 3G); although, the imaging of a 19 day sample shows traces of assembled structures comparable to those found at 21 day sample of B.

**Fig. 4:**
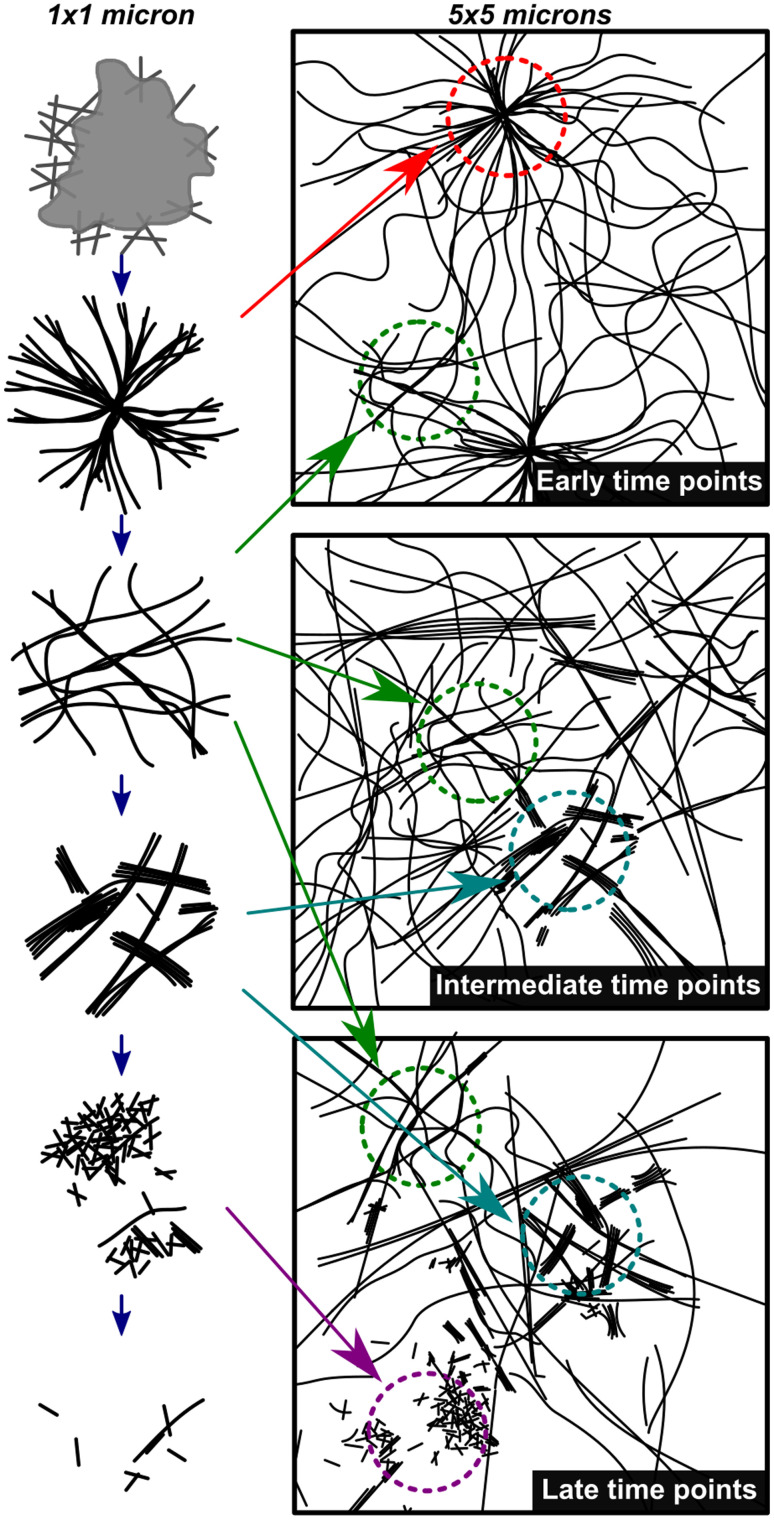
Mechanistic depiction of the synchronous process over time. The left column represents structures observed on a 1 × 1 micron scale (high magnification) and the right column is a representation of an overall sample (5 × 5 micron scale) highlighting the heterogeneity of fiber states observed. The depictions in the left column are representative of the cryoEM images in Fig. 3B–G in chronological order.

Fig. 4 shows a summary of the structures and general mechanistic trend in S. The mechanism is suggested based on the emergence and prevalence of different structural features from time-resolved cryoEM data (Fig. 3B–G), but we stress that each time point sampled contains mixed morphologies, notably those found in Fig. 3C–F. To illustrate this mechanism, Fig. 4 also shows a low magnification representation of an early time point where assembly is largely dominant (under 600 s), a middle time point, where both assembly and disassembly are prevalent (600–4300 s), and a late time point where disassembly is dominant (4300–23 400 s). The early time point representation shows a mix of the centrosome-like structures and dispersed fibers. The intermediate time point representation contains multidirectional flexible fibers as well as fibers and rods displaying stacking behavior. The late time points contain more stacked and disordered rods but still include nanorods that display some stacking.

**Fig. 5:**
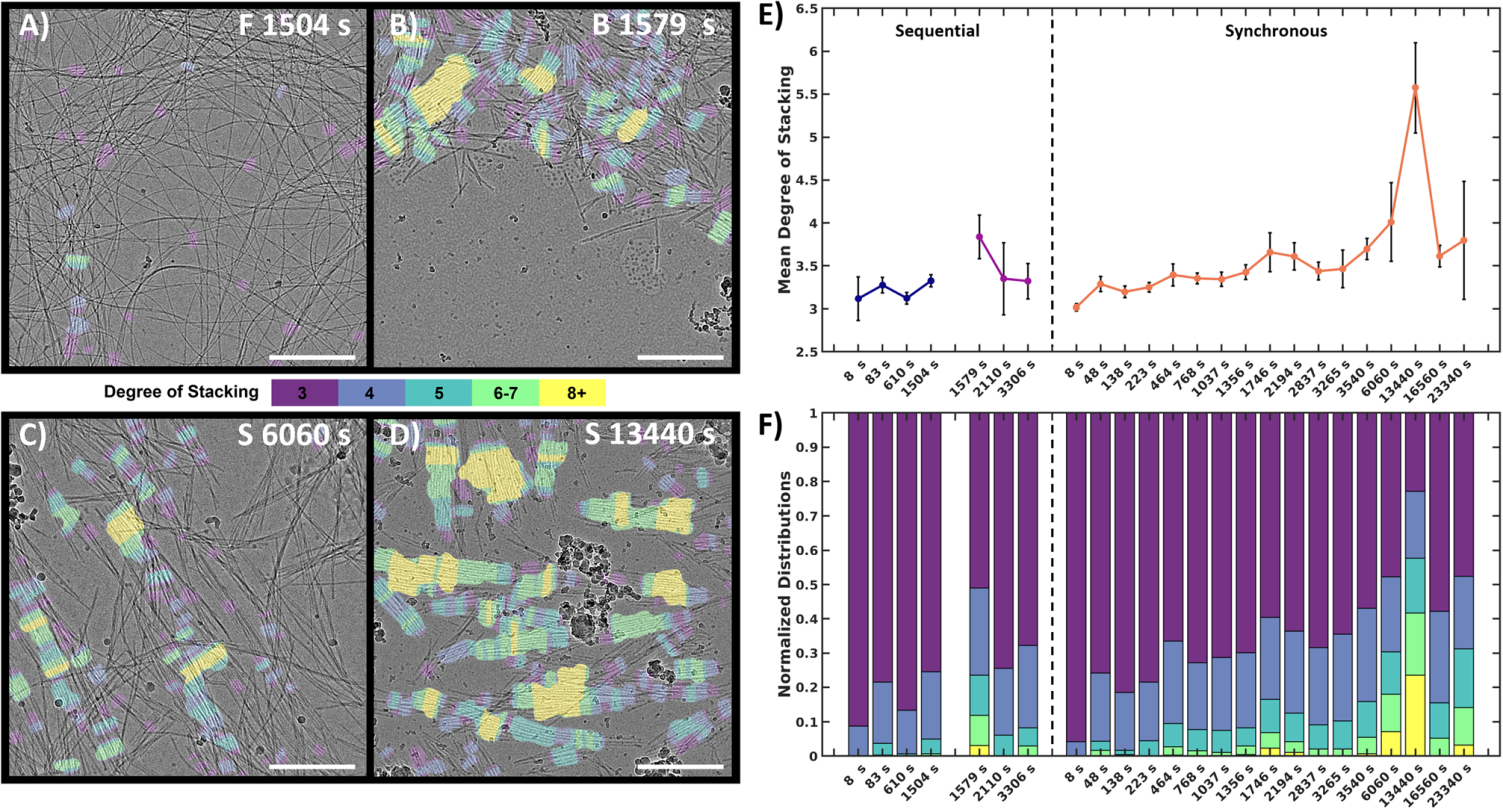
Quantitative image analysis of the stacked fiber phase. (A–D) Selection of labeled frames from key timepoints. Colored tinting corresponds to the degree of stacking. F = forward, B = backward, S = synchronous. (E) Mean DoS for each timepoint, high values indicate higher order in the system. Black error bars represent 95% confidence interval of mean, (details in ESI†). (F) DoS datapoint distribution normalized to 1 for comparison between timepoint. Scale bars 300 nm.

To quantify and track the fiber stacking, we developed an in-house MATLAB script to segment and analyze the stacked fiber phase (Fig. 5, details in Image processing section of ESI†). Stacked fiber domains were identified by first producing normalized cross-correlation maps using 108 synthetic templates to select for the stacked phase at different fiber spacings and angles in each images (Fig. S15†). This was possible because all cryoEM images were captured at the same nominal magnification (30k), so the pixel size of the fiber structures was consistent. It was found that the variation in microscope defocus impacted the normalized cross-correlation, so cross-correlation map thresholding was adjusted depending on the defocus of each image, which was estimated *via* radial integration of the fast-Fourier transform (Fig. S17†). Finally, fibers within the segmented domains were identified and labeled with the number of adjacent fibers, defined as the degree of stacking (DoS) (Fig. 5A–D and S18†). The image analysis pipeline was used to analyze 770 cryoEM images across 24 experimental conditions generating 398 million data points (Fig. S19–S21†). The resulting analysis tracks the structural evolution of the system by temporally quantifying the mean DoS (Fig. 5E), DoS distribution (Fig. 5F), and percent coverage (Fig. S21†) present at each timepoint.

The analysis reveals that both the sequential and synchronous processes show the presence of the stacked fiber phase, but exhibit different temporal behavior. In the sequential study, the forward process shows a low mean DoS with distributions dominated by low-DoS (≤4), which comes from the random distribution of fiber locations and relatively weak inter-fiber interactions (Fig. 5A). In contrast, the first timepoint in the backward reaction shows a sharp increase in mean DoS, indicating a substantial increase in inter-fiber interactions (Fig. 5B and E). The later timepoints show the system returning to a low DoS distribution, supportive of the previous qualitative observations that the high-DoS (≥5) stacked fiber phase is transient (Fig. 5E and F).

In the synchronous process, the mean DoS plot shows a gradual shift towards higher DoS structures during the first hour. The 6060 s and 13 440 s timepoints (Fig. 5C and D) show a significant increase in 8+ DoS (Fig. 5F and S21†), and the 13 440 s timepoint shows the highest mean DoS for all samples analyzed (Fig. 5E). This observation suggests that certain conditions can be exploited to control the order and amount of stacked fiber phase present in the system. Later timepoints reveal a drop in stacked phase density (Fig. S21†), while some high DoS domains persist (Fig. 5F). The data shows that the high-DoS structures are present for a period of >6 hours, which is substantially longer than the <600 s lifetime observed in the sequential system. The data also suggests that evolution of the stacked phase is a complex process. Fig. 5E shows subtle increases and decreases in the synchronous data which may indicate the amount stacked fiber phase present in the system is oscillating.

To provide insight into the chemical and physical mechanisms behind the differences between a sequential and synchronous process, we used simulations of the stochastic kinetics for both the chemistry and (dis)assembly in small, well-mixed volume elements (see ESI for more details on simulation: setup, rate constant determination, additional results†).^34^ A kinetic model of eleven reactions was devised, including the redox reactions driving the reaction network^23^ and the kinetics of fiber assembly and disassembly^39^ (Table 2). For each reaction, counts of the chemical species and fibers were simulated in time with kinetic Monte Carlo to account for the stochastic nature of the fiber growth and decay. These counts were recorded every 0.1 s for the forward, backward, and synchronous processes with the initial conditions matching the experiments. In addition to the evolution of concentrations, these simulations gave statistics for the number of fibers and their length. Once there is at least one fiber formed *via* reaction 4, the addition and removal of subunits to/from fibers has finite probability. Each time this reaction is selected to occur, a fiber is chosen at random to gain or lose one subunit.

**Table 2:** Chemical reactions of CSH/CSSC systems used to simulate the stochastic kinetics

Number	Reaction	Forward rate constant	Reverse rate constant
1	CSH + OH^−^ ⇌ CS^−^ + H_2_O	*k* ^CSH^_f_ = 10^pH−p*K*_a_^*k*^CSH^_r_	*k* ^CSH^_r_ = *rD*(CSH)/*V*
2	CS^−^ + H_2_O_2_ → CSOH + OH^−^	*k*_3_ = 25 M^−1^ s^−1^	
3	CS^−^ + CSOH → CSSC_sol_ + OH^−^	*k*_4_ = 720 M^−1^ s^−1^ [4]	
4	CSSC_sol_ ⇌ CSSC_fib_	*k*_7_ = 1.46 × 10^−17^ M^−1^ s^−1^	*k*_sp_ = 1000 M^−1^ s^−1^
5	DTT + OH^−^ ⇌ DTT^−^ + H_2_O	*k* ^DTT^_f_ = 10^pH−p*K*_a_^*k*^DTT^_r_	*k* ^DTT^_r_ = *rD*(DTT)/*V*
6	DTT^−^ + CSSC_sol_ → DTTSC + CS^−^	*k*_6_ = 5 M^−1^ s^−1^	
7	DTT^−^ + CSOH → DTTSC + OH^−^	*k*_5_ = 10 M^−1^ s^−1^	
8	DTTSC → DTT_cyc_ + CSH	*k*_d_ = 2.83 × 10^−17^ M^−1^ s^−1^	
9	DTT^−^ + H_2_O_2_ → DTTOH + OH^−^	*k*_1_ = 0.0046 M^−1^ s^−1^	
10	DTTOH → DTT_cyc_ + OH^−^	*k*_d_ = 2.83 × 10^−17^ M^−1^ s^−1^	
11	2CSSC_sol_ → 2CSSC_fib_	*k*_7_ = 1.46 × 10^−17^ M^−1^ s^−1^	

Most rate constants in the chemical mechanism have been measured experimentally (Simulation section of ESI†). Rate constants of diffusion-limited reactions were taken to be the diffusion coefficient of the relevant species.^35^ The remaining unknown rate constants were optimized to match experimental timescales. Using F as a starting point, a range of rate constants for (*k*_sp_, *k*_3_, and *k*_6_) that gave agreement were further narrowed down to ensure agreement with the observed timescale of B. For these steps, *k*_sp_ and *k*_6_ were varied because they had not been previously experimentally determined, and *k*_3_ was varied because the experimental value did not result in fiber growth in the simulations (see Simulation ESI for detailed explanation of rate constant determination†). Here, the forward process involves reactions 1–4 and 11 and the backward involves 4–8 and, to a lesser extent, 9–10 with any remaining H_2_O_2_. In the synchronous process, all reactions in the chemical mechanism in Table 2 can occur. Using the parameters and ranges from the sequential process, the timescale of the simulated synchronous process was then optimized to be as close to the experimental timescale as possible by varying the rate constants unimportant to F and B or still having a range of possible values. This procedure gave simulation timescales that agreed with those observed for F and B within an acceptable error and a timescale of ∼22 535 s in agreement with the timescale for S.

**Fig. 6:**
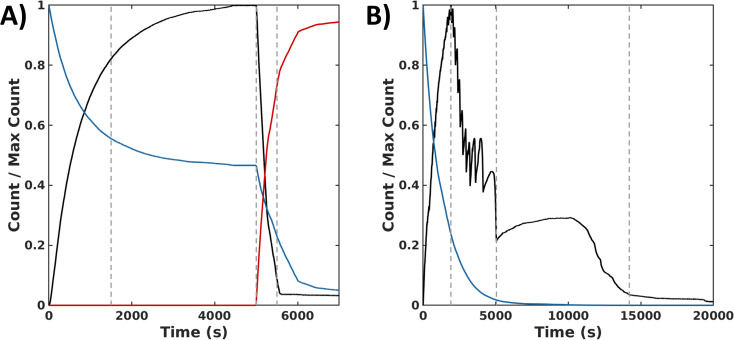
Simulation data for the sequential and the synchronous processes. (A) Normalized time series of species count for the forward (0 s ≤ *t* ≤ 5000 s) and backward (*t* > 5000 s) processes reported in Δ*t* = 0.1 s time intervals: CSSC in fibers (black), H_2_O_2_ (blue), and DTT_cyc_ (red). 1^st^ dashed line indicates >75% completion of the forward reaction. 2^nd^ dashed line indicates the addition of DTT which is then converted to DTT_cyc_. 3^rd^ dashed line represents >75% completion of the backward reaction. (B) Normalized count of assembled subunits CSSC_fib_ (black) and oxidant H_2_O_2_ (blue) in time reported in Δ*t* = 0.1 s time intervals. 1^st^ dashed vertical line indicates time of peak amount of CSSC incorporated into fibers. 2^nd^ dashed line marks the end of the oscillatory stage. 3^rd^ dashed line marks the near depletion of CSSC fibers.

In the sequential process, the simulations show a rapid conversion of CSH to an intermediate CSOH, which resolved to CSSC during F. With an accumulation of CSSC, rapid fiber growth leads to steady values of both fiber count and length (average 35 units) by 4000 s. To start the backward process, DTT was injected at 5000 s. The presence of this reductant causes the CSSC count to quickly fall through the conversion of DTT to the cyclic form of DTT (DTT_cyc_) (Fig. 6A) leading to the fast degradation of fibers. The timescale of these simulations matches the magnitude of the experimental timescale. Simulations of the synchronous process also show an initial rapid buildup of CSSC and fibers within the first several minutes (2500 s). Following this initial buildup of CSSC, fiber counts then oscillate while also steadily decaying. Around 5000 s, when the oxidant is roughly depleted, the value of CSSC plateaus until a final decrease to trace counts of CSSC around (15 000 s) (Fig. 6B). The fibers for the synchronous process are shorter (30 units) than the forward process (35 units). Even accounting for the concentration dependence of rate constants, the timescale of the simulations is about 20 000 s (∼5.6 h) which is shorter than the experimental results (∼8 h), suggesting the importance of the kinetics of diffusion and fiber growth. Analyzing these simulation data, we identified a sequence of chemical reactions (fiber growth through reactions 2, 3 and fiber decay through reactions 6 and 8) that occurs approximately 10 times more often in the synchronous process than in the sequential process (see Simulation section of ESI†).

## Discussion

Collectively, the experimental and computational data shows that the synchronous system is not simply a combination of a sequential assembly and disassembly process. For example, the time scale of the synchronous process (∼8 h) is substantially longer than the sequential forward and backward processes (<1 h). One reason is that a synchronous process can locally cycle between assembly and disassembly until the excess H_2_O_2_ is depleted. This cycling increases the total number of reactions that occur in the synchronous process than the sequential process. The sequential process cannot repeatedly execute this cycle and the amount of H_2_O_2_ and DTT used is limited by the initial concentration of CSH (10 mM). However, the cycle alone is likely not sufficient to explain the disparity in the time scale of the synchronous and sequential process. The simulations, which account for the cycling and stochasticity of the reaction and fiber growth kinetics, predict the synchronous process to be ∼20 000 s (5.6 h) and the sequential process to be ∼5000 s (1.4 h). They also suggest that accurately modeling the transient structures formed by synchronous reactions requires not only detailed modeling of chemical kinetics but also the diffusive kinetics and concentration gradients across longer length scales. This reasoning is consistent with previous work showing a time delay results between the chemically driven reaction and the self-assembly.^36^ This discrepancy could account for the difference between the experimental and simulation timescales of the synchronous process. Additionally, local diffusion gradients are likely one reason why the synchronous system we observe is more complex and heterogeneous compared to the sequential process. The simulations show an oscillatory behavior in the fiber count that we attribute to a subset of reactions forming a cycle; there is also a maximum in the count of CSSC in fibers followed by a quick collapse. Similarly, the quantification of the stacked phase in the cryoEM data shows a complex evolution in the normalized density distribution followed by a maximum and collapse. This suggests that the complex evolution could be the result of an underlying oscillator behavior. While there are different mechanisms capable of generating oscillations,^37,38^ our simulation data suggest the repeated occurrence of a subset of reactions that form a cycle are responsible for the oscillations. Experimental confirmation for our system would require *in situ* imaging with nanoscale resolution. The most important point here is that in both the simulations and the cryoEM data,^31^ the morphology evolution in the synchronous system is much more complex than the sequential process.^39^

The sequential and synchronous processes are most similar in the early and late time points, when the assembly and disassembly reactions respectively dominate. For example, both the synchronous and sequential processes begin with the formation of large (>1 micron diameter) unstructured aggregates. Such aggregates, which are known as liquid or solid phase precursors, are very common in fiber formation and crystallization processes.^40–42^ These precursors phases contain high local concentration of building blocks and can result in rapid fiber nucleation.^41–43^ For example, microtubules, which grow as supramolecular fibers, have been shown to form *via* a liquid droplet precursor mechanism.^44,45^ These liquid precursors convert into centrosomes, which are the organization centers for microtubule growth.^45,46^ We postulate that the high-density fiber regions we observe are analogous to these organization centers and we refer therefore to them as centrosome-like structures (Fig. 1B, 3B and C). Interestingly these centrosome-like structures appear in both the sequential and synchronous processes indicating they are part of a general feature of fiber formation rather than a specific feature of the synchronous chemistry. At the late time points both processes show trace amounts of assembled structures either due to kinetic trapping or residual oxidation of CSH.

The most distinctive difference between the sequential and synchronous processes are seen in the intermediate time points when the kinetics of both the forward and backward processes are sufficiently high to influence the morphology of the assemblies. During the forward reaction, the sequential process rapidly forms a thermodynamically stable product (Fig. 1A, C and D). In the backward disassembly step, as DTT is added, the fibers become unstable, form a transient intermediate stacked phase which rapidly disassembles (Fig. 2A and C). During the synchronous process the constant cycling, along with factors mentioned above, enables the stacked phase to be sustained for ∼6 hours and is present in combination with disperse fibers (Fig. 3A, E and S13†). The cryoEM images of the stacked phase in the synchronous process show instances of single fibers that are partially isolated and partially in a stack (Fig. S22†). This observation suggests stacks likely form from the aggregation of individual isolated fibers, rather than the stacked phase growing independently from the isolated fibers. In the backward reaction this likely occurs as the long fibers become shorter due to disassembly. In the forward reaction, once the fibers reach a certain length it is not entropically favorable for large stacks to form. Importantly, the stacked phase in the backward reaction is limited to short rods whereas in the synchronous process, both fibers and rods can show ordered stacking. This indicates that in addition to the synchronous process stabilizing a transient intermediate that was observed in the sequential process, it is also capable of producing structures with order over larger distances *e.g.*, stacked fibers. Consequently, we believe the origin of the sustained stacking in the synchronous system is likely due to the ability of the synchronous assembly/disassembly to dynamically modulate the length of the fibers to favor entropically driven aggregation. However, we cannot rule out other mechanisms that could be related to the synchronous chemistry altering the molecular organization of the fibers.

For the synchronous process the degree of stacking reaches a maximum in later time points (Fig. 5E), which coincides with a period of sustained fiber count in the simulations when the oxidant is almost depleted. From this data, a plausible design principle emerges from the nonlinear nature of the kinetics: smaller amounts of oxidant might be necessary to sustain the fibers compared to the amount of oxidant needed to create them initially. To test this hypothesis, we simulated the addition of small doses of H_2_O_2_ at regular intervals in the synchronous process to sustain the fiber population (see Simulation section of ESI†). The simulations show that fibers can indeed be sustained with a small amount of oxidant when there is feedback between fiber structures and added oxidant. More work is needed to fully exploit this principle; however, we believe that minimal oxidant (or reagent) doping could be a promising method to maintain highly ordered thermodynamically unstable structures. We think this could be an important way forward for the field as a similar approach has proven highly beneficial in the inorganic nanocrystal community where studies of transient species formed through nonequilibrium chemistry has enabled the trapping of nanostructures with exceptional properties.^47–50^ Although the chemistry and applications for these supramolecular systems will be different from inorganic nanocrystals, we believe the general principle, that thermodynamically unstable structure can have different and potentially exceptional properties in comparison to thermodynamically stable structures should be true for both classes of materials.

## Conclusion

In summary, we have compared the self-assembly mechanisms for a conventional sequential assembly–disassembly process and a synchronous process using time-resolved cryoEM, quantitative image analysis, and kinetic Monte Carlo simulations. Here, by isolating and studying the sequential process, we identify which morphologies are thermodynamically stable and which are thermodynamically unstable. In the synchronous process, we show that early and late timepoints of the system resemble that of a conventional assembly process. During the middle stages of the synchronous reaction, the samples display structural features also found in the sequential process. However, each time point in the synchronous process is more heterogeneous and the evolution of structures is more complex. Importantly, we show that transient, yet highly ordered, intermediates in the sequential self-assembly process can be stabilized using synchronous chemistry. Simulations were used to rationalize these differences, suggesting they may arise from less diffusion and shorter fiber lengths in the synchronous process compared to the sequential process. From a design point of view, these findings are important as we can now tune the reaction kinetics to select for and enhance the yield of this unstable phase. We anticipate that these findings will also be useful for understanding how higher-ordered systems are maintained using synchronous chemistry and will aid in establishing tunable structure–property relationships and subsequent applications.

## Data availability

Raw electron microscopy data and the code for the data analysis is available from JPP upon reasonable request. Data and code for the stochastic simulations of the chemical kinetics are available from JRG upon reasonable request.

## Author contributions

JPP conceived and supervised the study. PJH carried out cryoEM experiments. JTM and RFH performed the image analysis. JRG and RAB conceived and performed computational studies. PJH, ZG and SS determined the experimental parameters for data collection. JPP, PJH, and JTM and wrote the manuscript. JPP, JRG, and ZG supervised the study. PJH, JTM, and RFH co-wrote the ESI.† All authors reviewed and edited the manuscript.

## Conflicts of interest

The authors declare no competing financial interest.

## Supplementary Material

SC-015-D3SC05790A-s001
