# Supplementary material for: CryoEM reveals the complex self-assembly of a chemically driven disulfide hydrogel
Source: Chem Sci. 2023 Dec 18;15(3):1106–16. doi: 10.1039/d3sc05790a (PMC10793653; doi:10.1039/d3sc05790a)
Supplement: SC-015-D3SC05790A-s001 [file SC-015-D3SC05790A-s001.pdf]

Electronic Supplementary Material (ESI) for Chemical Science.
This journal is © The Royal Society of Chemistry 2023

Supplementary Information for

CryoEM Reveals the Complex Self-assembly of a Chemically Driven Disulfide Hydrogel

Paul Joshua Hurst[1,2]; Justin T. Mulvey[2,3]; Rebecca A. Bone[4]; Serxho Selmani[1,2]; Redford F. Hudson[5]; Zhibin Guan[1-3,6-7]; Jason R. Green[4,8]; Joseph P. Patterson[1-3]*

Affiliations

1. Department of Chemistry, University of California, Irvine, Irvine, California 92697, United States
2. Center for Complex and Active Materials, University of California, Irvine, Irvine, California 92697, United States
3. Department of Materials Science and Engineering, University of California, Irvine, Irvine, California 92697, United States
4. Department of Chemistry, University of Massachusetts Boston, Boston, Massachusetts 02125, United States
5. Department of Computer Science, University of California, Irvine, Irvine, California 92697, United States
6. Department of Chemical and Biomolecular Engineering, University of California, Irvine, Irvine, California 92697, United States
7. Department of Biomedical Engineering, University of California, Irvine, Irvine, California 92697, United States
8. Department of Physics, University of Massachusetts Boston, Boston, Massachusetts 02125, United States

Table of Contents

Sections

Experimental/Cryogenic transmission electron microscopy ---2-15

Image processing and analysis ---15-22

Simulations ---23-37

References ---38

Experimental/Cryogenic transmission electron microscopy section

Table S1: Experimental conditions for the sequential process as well as controls.

Run*	[CSH/CSSC] mM	[H_2O_2] mM	[DTT] mM	# Time Points
**F**	10	10	n/a	4
**F-1C**	10	150	n/a	3
**F-2C**	4	150	n/a	1
**B**	10	10	200	4
**B-1C**	10	5	200	1

Experimental Controls:

According to the kinetic data from the previous CSSC study, CSSC reaches a maximum conversion of 40%. The reaction cannot reach 100% because of the presence of DTT. Therefore, to understand the nature of CSSC at lower conversions and indicate if CSH (the inactive building block) participates in the assembled structures, we focus our study on **F** with one equivalent of H_2O_2 and CSH. Control experiments were performed by running the forward reaction rapidly to 100% conversion with excess H_2O_2 as outlined in conditions **F-1C** and **F-2C** (Table S1, Figure S4-5). In **F-1C**, the amount of H_2O_2 and CSH is kept identical to the synchronous conditions which leads to rapid conversion. In **F-2C**, the amount of H_2O_2 is kept identical to the synchronous conditions but the amount of CSH is reduced to 4mM, thus achieving the same maximum concentration of CSSC as the synchronous conditions. These controls yield nanofibers similar to **F** (Figure S6-7)

The backward reaction was selected by taking **F** and treating it with 200 mM DTT, the same concentration of DTT in the synchronous process. This led to rapid degradation of CSSC back to CSH. However, due to trace amounts of H_2O_2 left in **F**, another forward reaction with 5 mM H_2O_2 was run to completion and DTT was subsequently added (**B-1C**). In this sample, the stacked phase was still visible at an early time point indicating that the trace H_2O_2 does not affect the morphology (Figure S10).

Lastly, we wanted to see if DTT impacted the morphologies we obtained due to the potential for dual hydrogen bonding, so we used an alternative reducing agent, dithiobutyl amine (DTBA), which is not capable of dual hydrogen bonding. We obtained similar types of fibers with DTBA (Figure S14). It should be noted that DTBA has differing kinetics to DTT.

Selection of CryoEM Parameters:

Preliminary studies were carried out to optimize all cryoEM parameters. During these optimization experiments we observed results consistent with the main data presented in this manuscript. A blot time of 3s gave a good ice layer. We did not use a post blot time. Post blot times are typically used to relax structures back to equilibrium following an application of shear force from blotting.[1,2] However, our system is out-of-equilibrium and is in constant flux and will not return to equilibrium during a post blot relaxation. For this reason, we believe that a post blot relaxation time would just result in the reorganization of structures within the confined environment of the thin water layers on the cryoEM grid which would not be representative of the bulk structure. Consequently, we believe that trapping the structures though vitrification as quickly as possible is the best preparation method for these samples. As this study is comparative, cryoEM parameters were kept the same for every sample and timepoint. It is also important to highlight that ,any well-known time-resolved cryoEM studies have also foregone post blot times.[3,4]

30k was selected for magnification as it is the highest magnification that does not cause visible beam damage of the sample using low dose imaging. This allows a large area of the sample to be inspected in a single image. On average, each timepoint had approximately 30 images collected. We opted to capture additional timepoints rather

than repeating the sample multiple times with a smaller number of time points. We were also sensitive to the image processing time and therefore had to find a balance between the number of images collected and time required for image processing. Our study alone, encompassing 770 images which were processed in parallel using the UCI HP3. Each image was allocated 30 cores, 100GB of RAM, and 1 hour of processing time. It took approximately 23,000 core-hours in total. Because of efficient parallelization, the time to complete the entire analysis was 5 hours for a given set of parameters. This processing collected 398 million data points with an average of over 500,000 per image with a very large standard deviation as images contain little to no stacking.

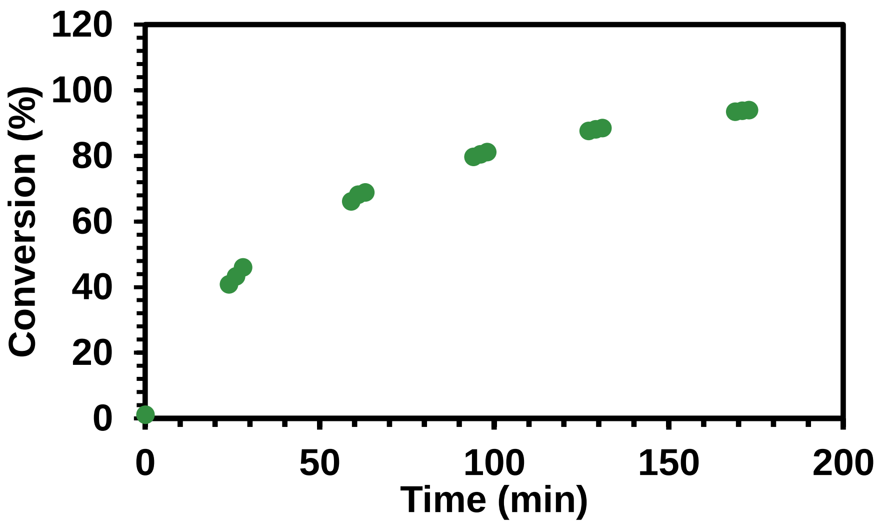

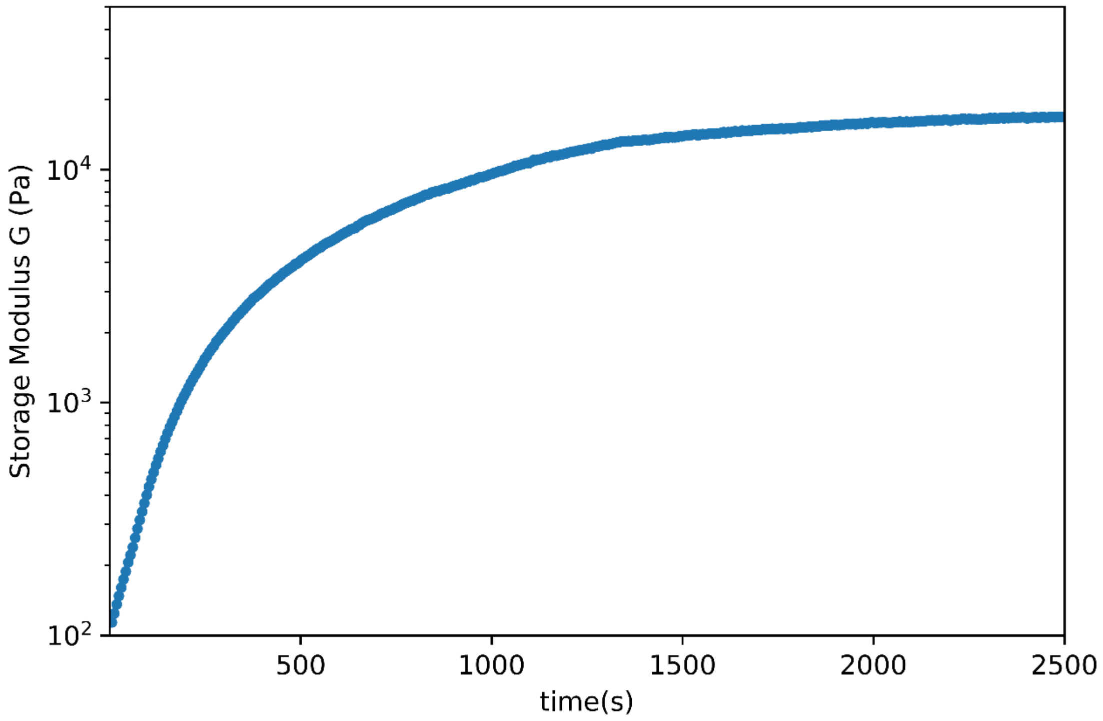

Figure S1: UPLC and rheology data for forward reaction. The rheology is of sample **F**. After about 30 minutes, the rheology data plateaus indicating a maximum development in gel strength. The HPLC data is of an analog of CSH that does not contain an aryl group which can explain the discrepancy between the rheology and UPLC data. The analog of CSH is used as it does not gelate. Gelation has clogged the UPLC columns, causing damage.

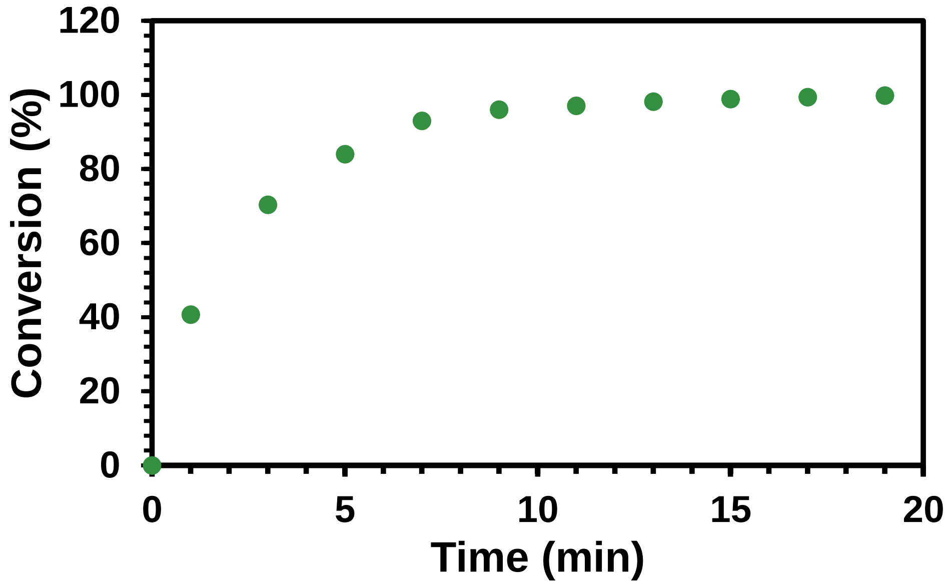

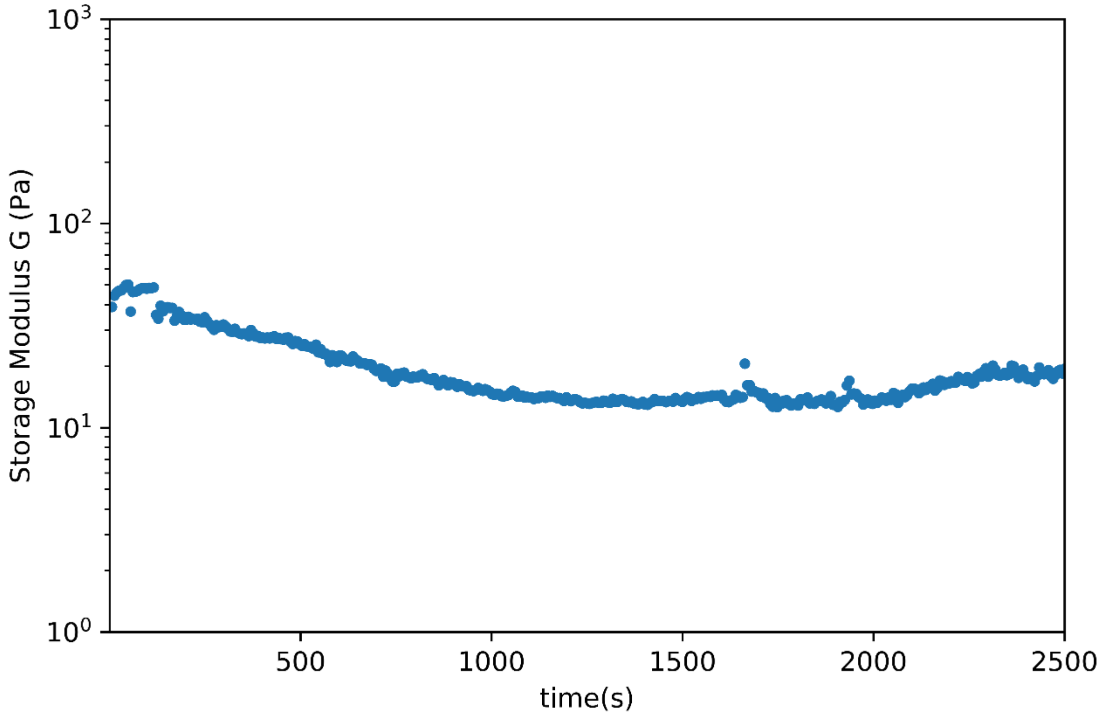

Figure S2: HPLC and rheology data for backward reaction. The HPLC data is of an analog of CSH that does not contain an aryl group. The rheology is of sample **B** and instantly de-gels following the addition of DTT.

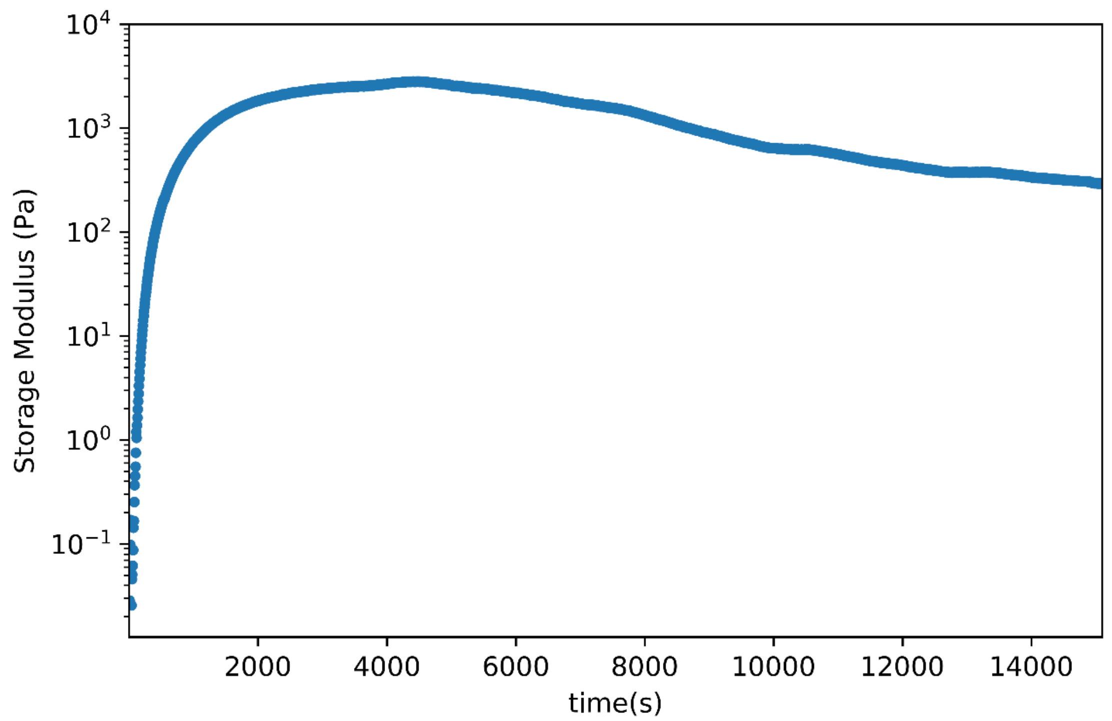

Figure S3: Rheology data for the synchronous process, **S**.

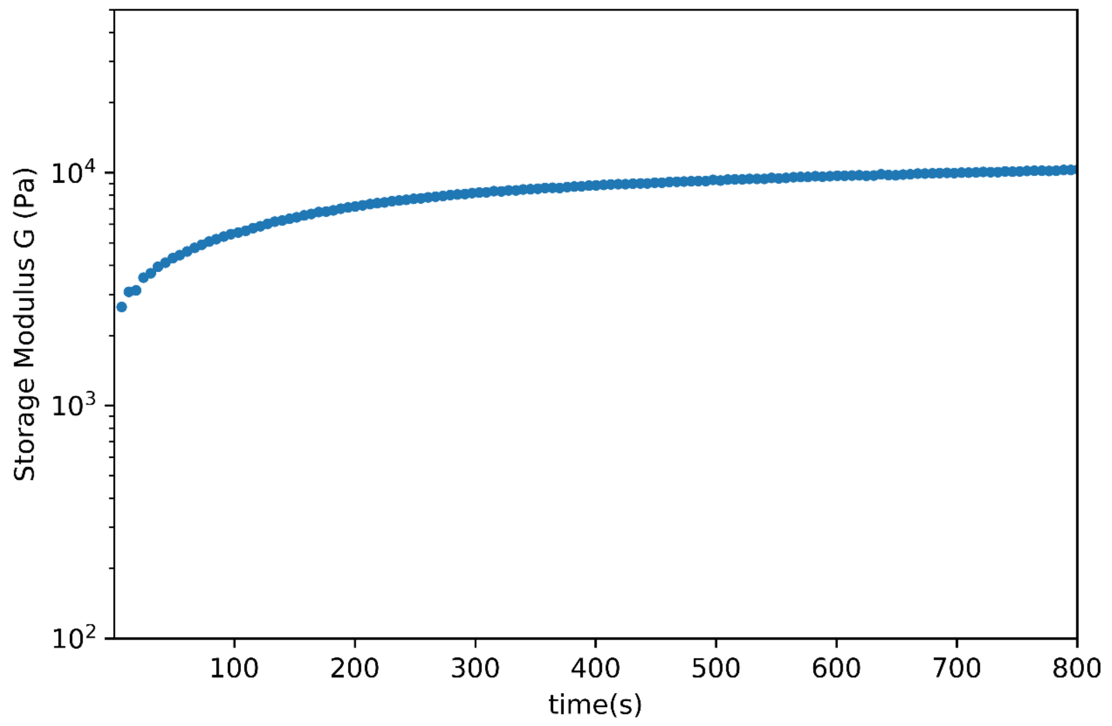

Figure S4: Rheology data for forward reaction **F-1C** with excess H_2O_2 at 10 mM CSH.

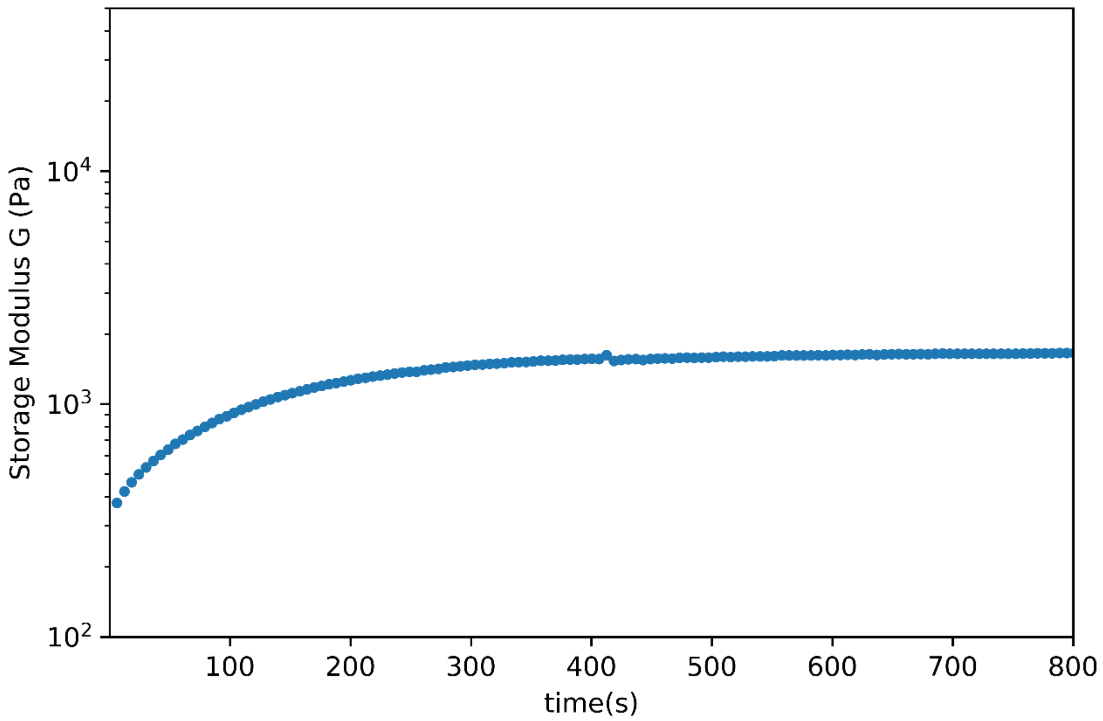

Figure S5: Rheology data for forward reaction **F-2C** with excess H_2O_2 at 4 mM CSH.

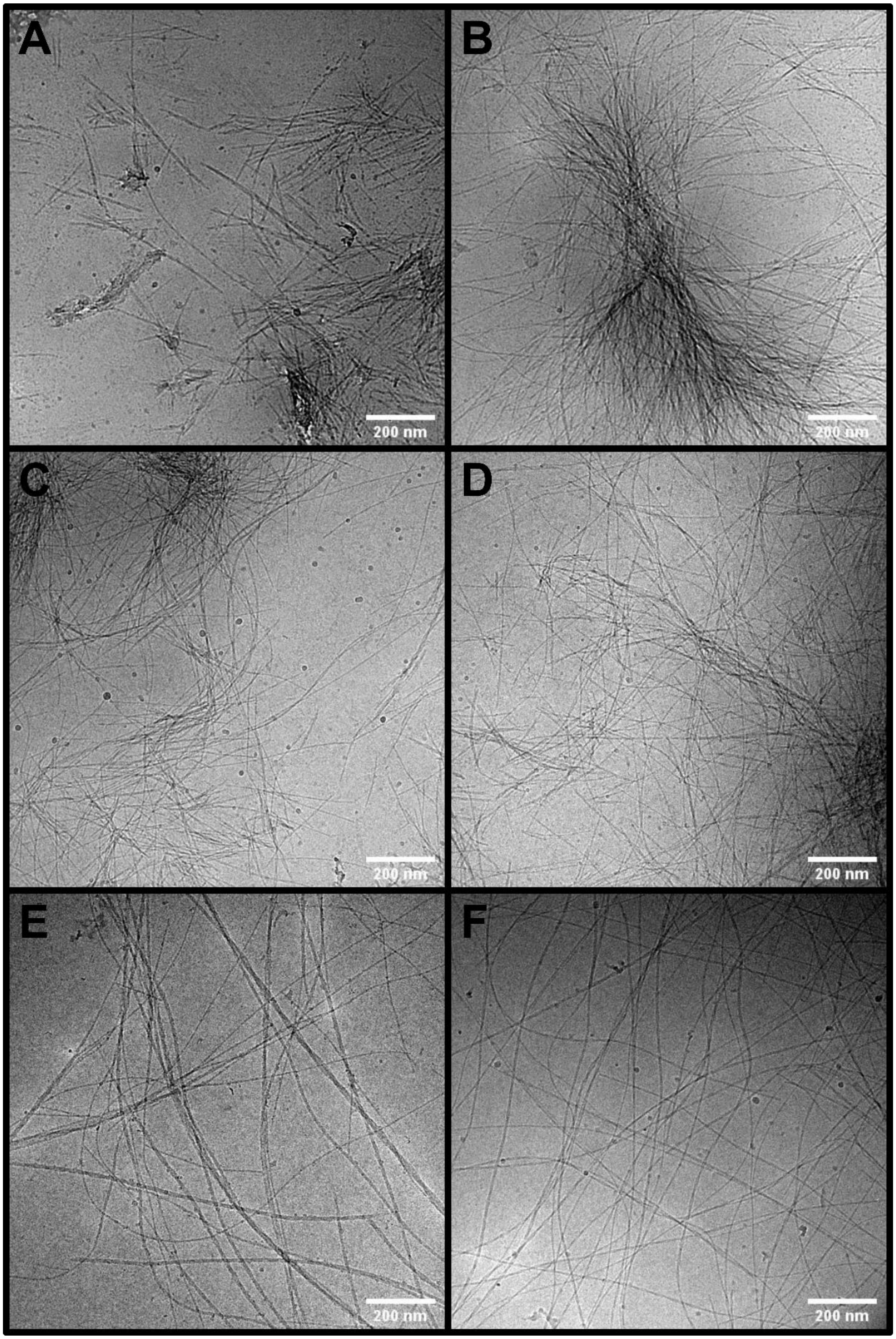

Figure S6: Time-resolved CryoEM images of **F-1C**. A-B) 8 s C-D) 55 s E-F) 10 min 3 s

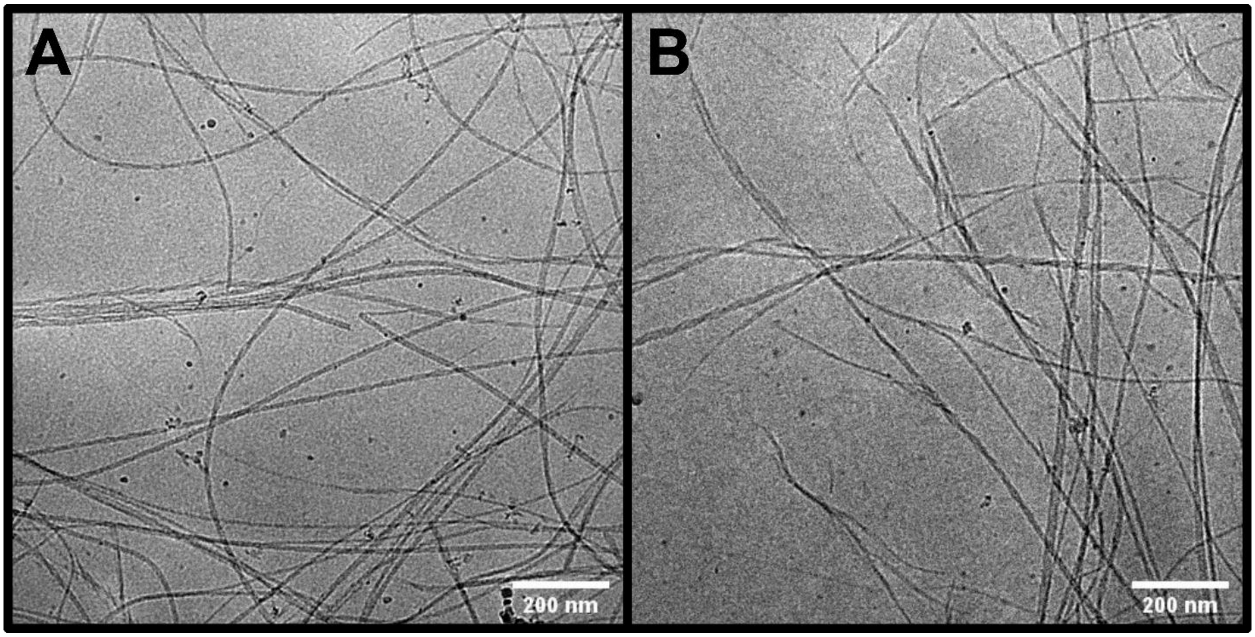

Figure S7: CryoEM images of **F-2C** taken at 2 min 15 s

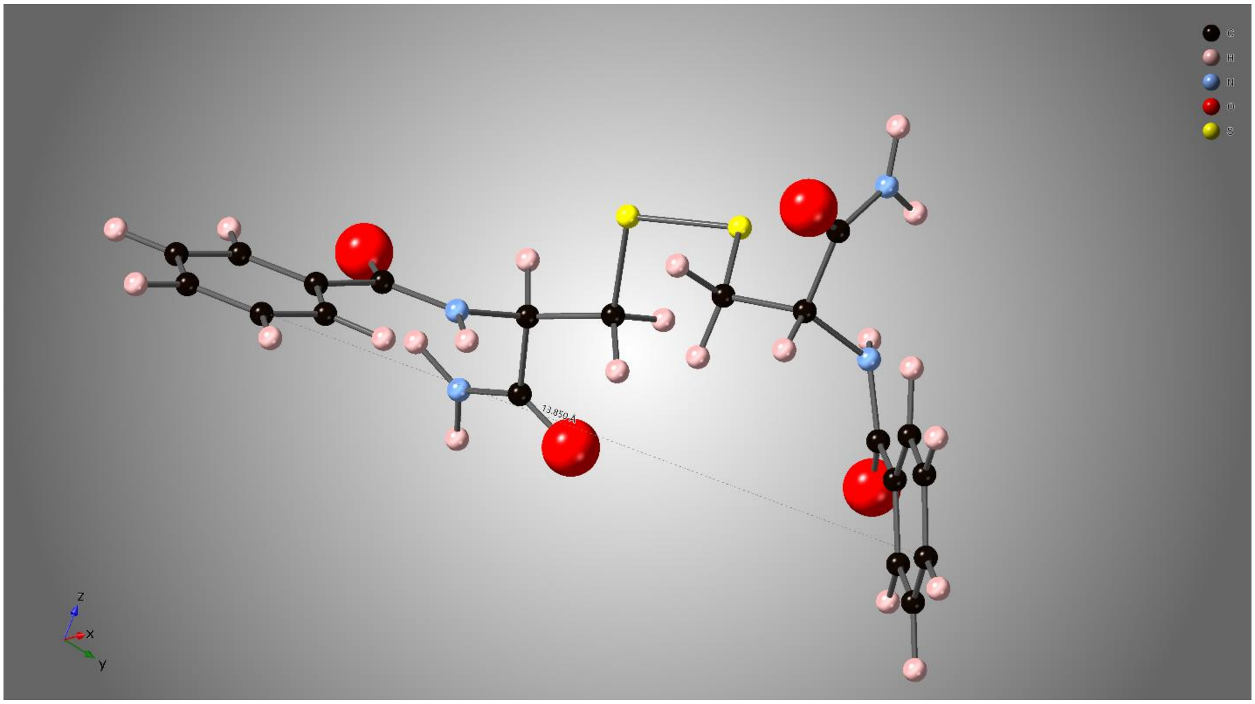

Figure S8: Representative CSSC molecule made in Crystal Maker. The distance between carbon 4 in the aryl rings is about 13.8 Å. The distance between the sulfur and the aryl groups is approximately 12 Å. This structure does not represent a solved crystal structure and is used to get an idea of molecular length only.

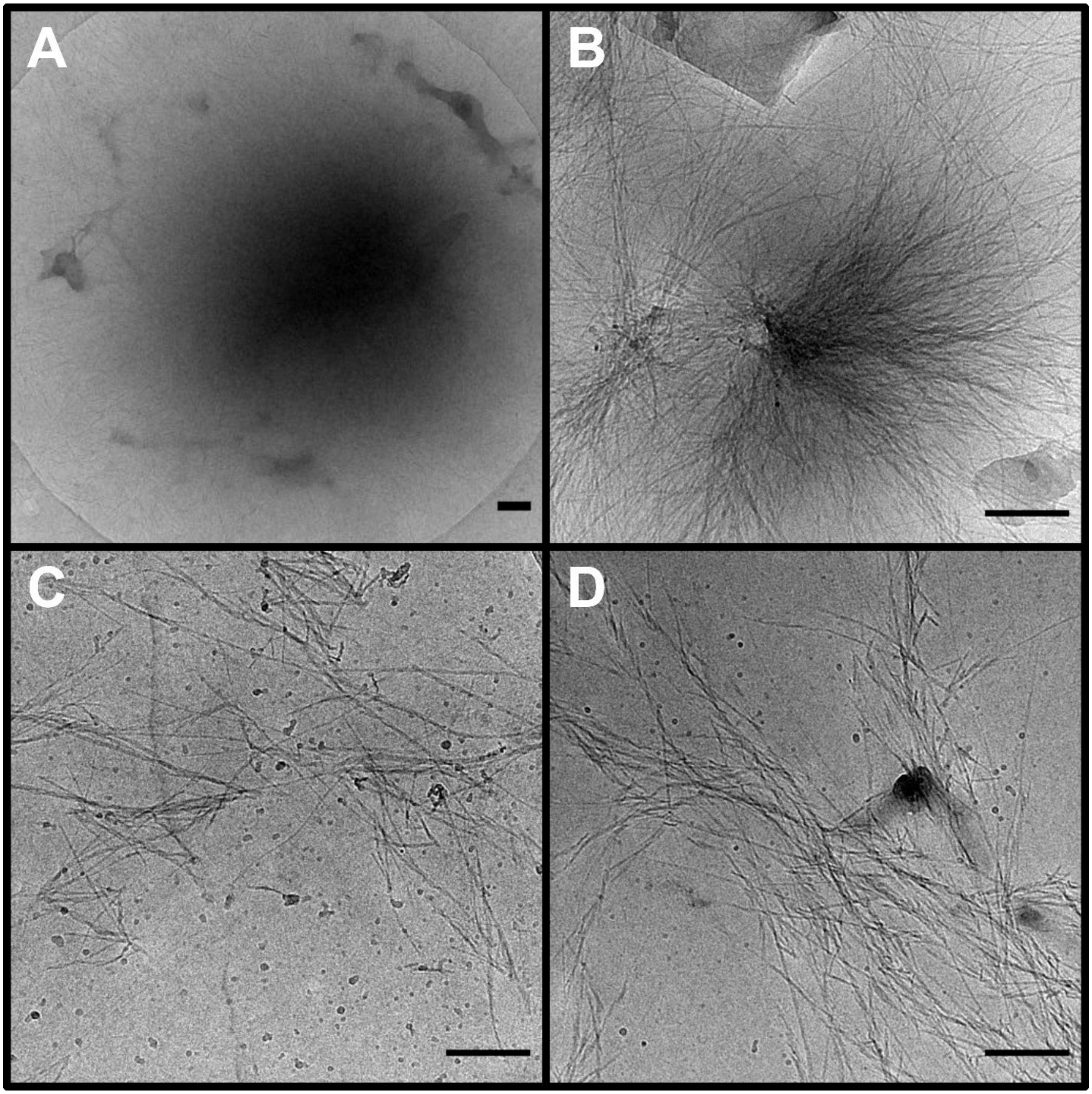

Figure S9: CryoEM images of **F** from 8 s. A shows what appears to be a liquid droplet precursor to fibers. B shows a centrosome-like structure whereas C and D show some fibers that do not appear to originate from a larger (micron-scaled) cluster. Scale bars are 200 nm.

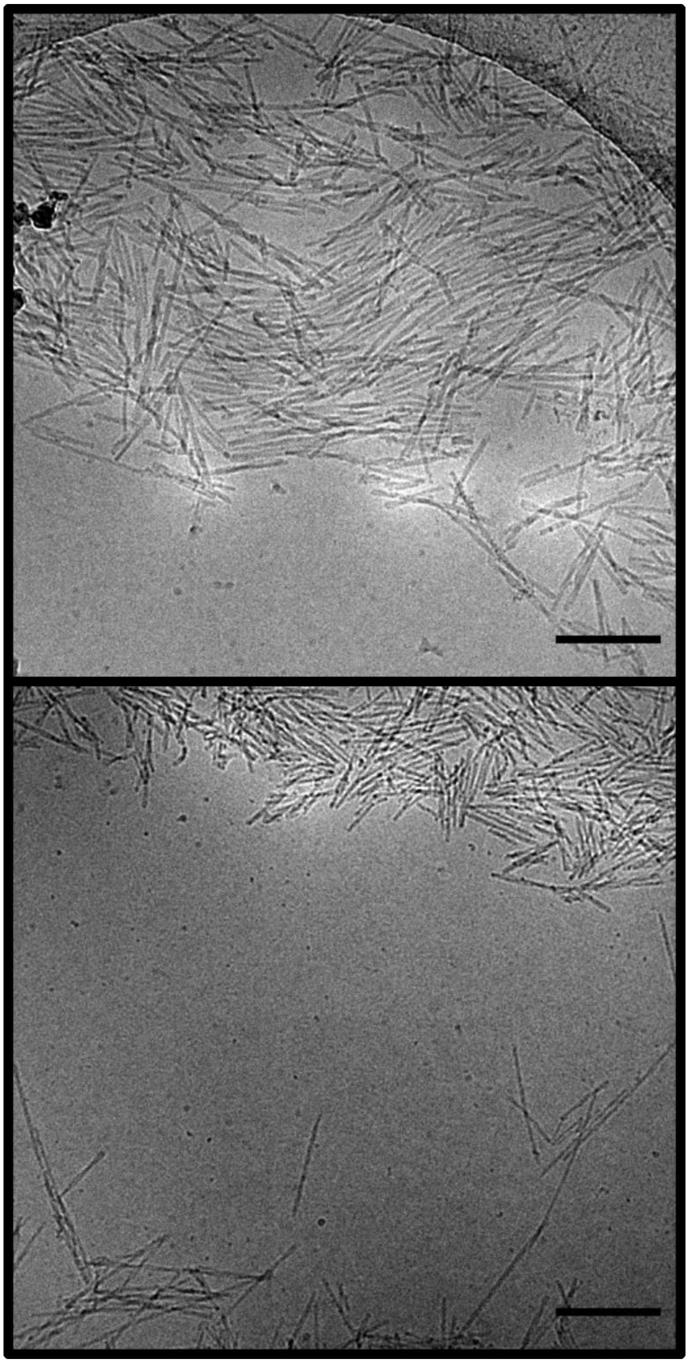

Figure S10: CryoEM images of **B-1C** at 44 s after adding DTT. The stacked phase is still present even though there is no residual fuel remaining (5 mM of H_2O_2 used to assemble 10 mM of CSH before the addition of 200 mM DTT).

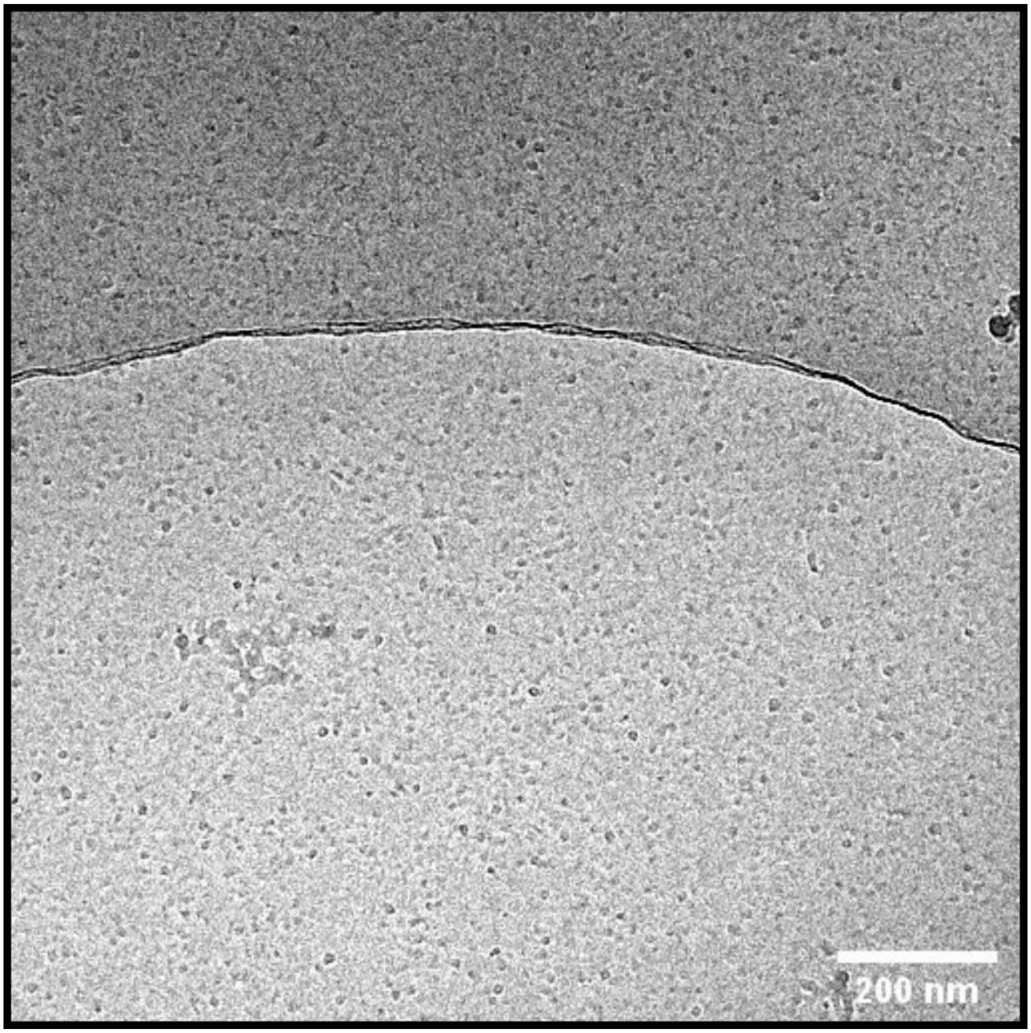

Figure S11: Representative cryoEM image for the t=0 timepoint for **S**. This timepoint is before the addition of H_2O_2. The black spheres represent ice contamination.

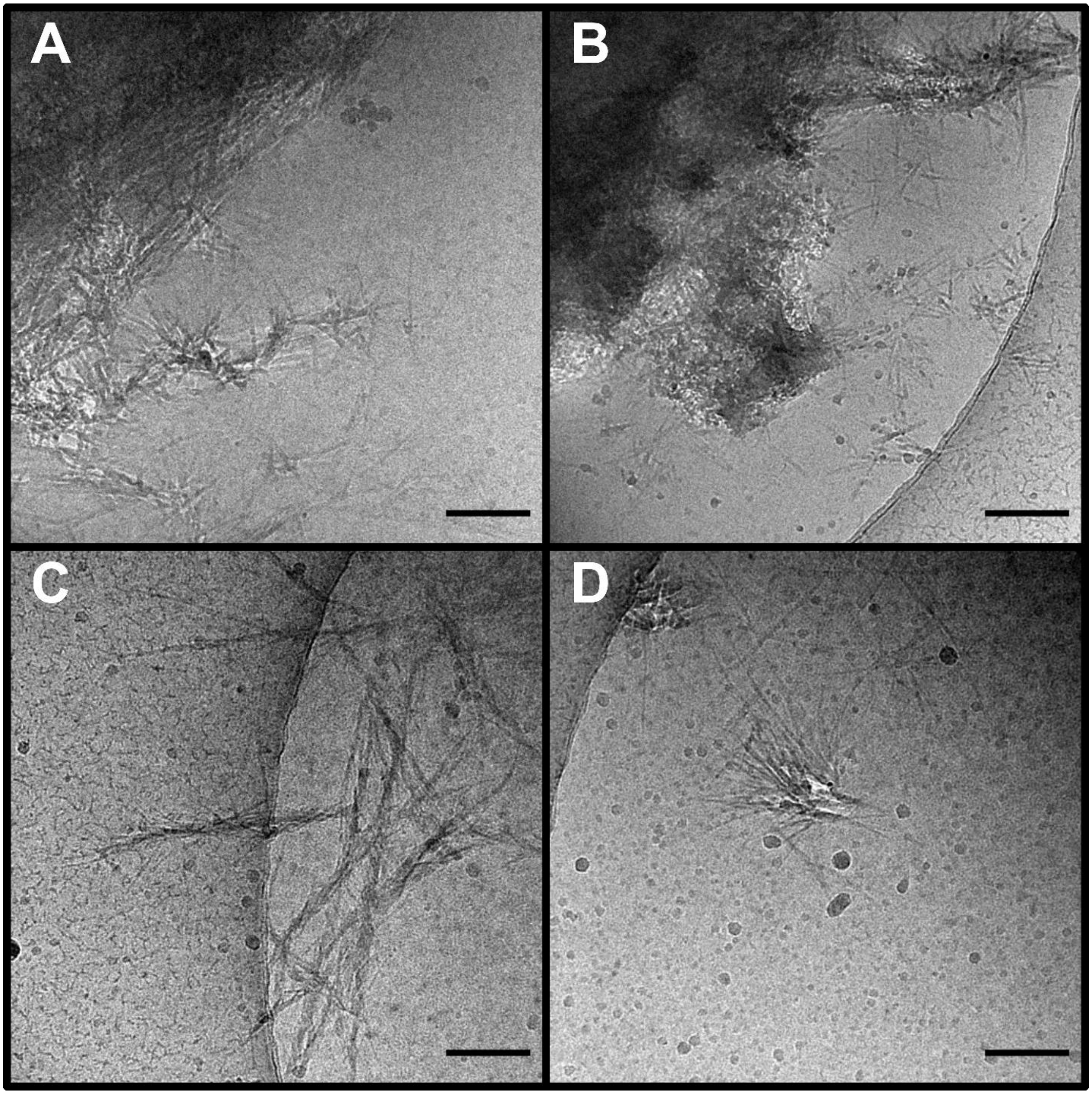

Figure S12: CryoEM images of **S** at t = 8s. A and B show liquid droplet precursors whereas C and D show more isolated structures that could have nucleated from unimer growth.

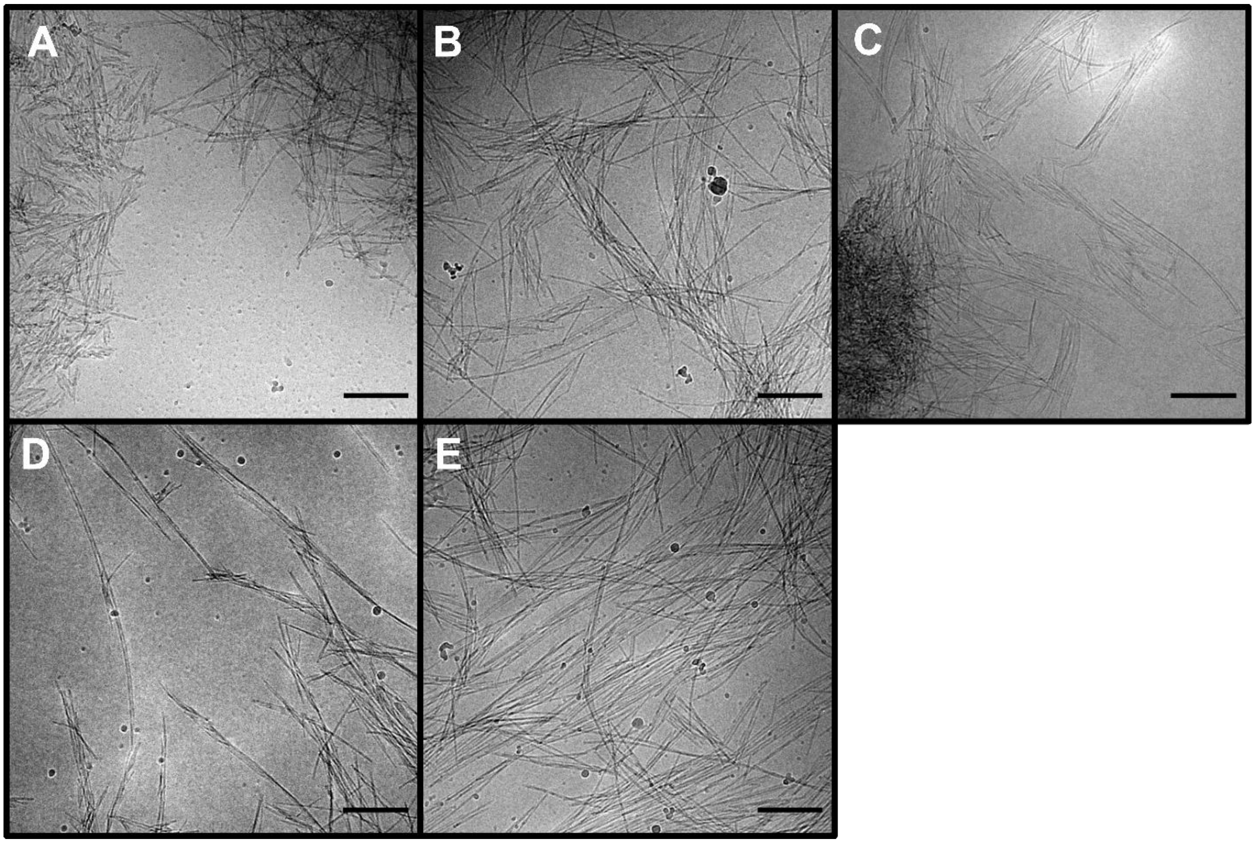

Figure S13: CryoEM images of **S** from multiple time points showing stacked phase. A) The first stacked phase was seen at 7 min 44 s B) 22 min 36 s C) 29 min 6 s D) 36 min 34 s E) 1 h 41 min

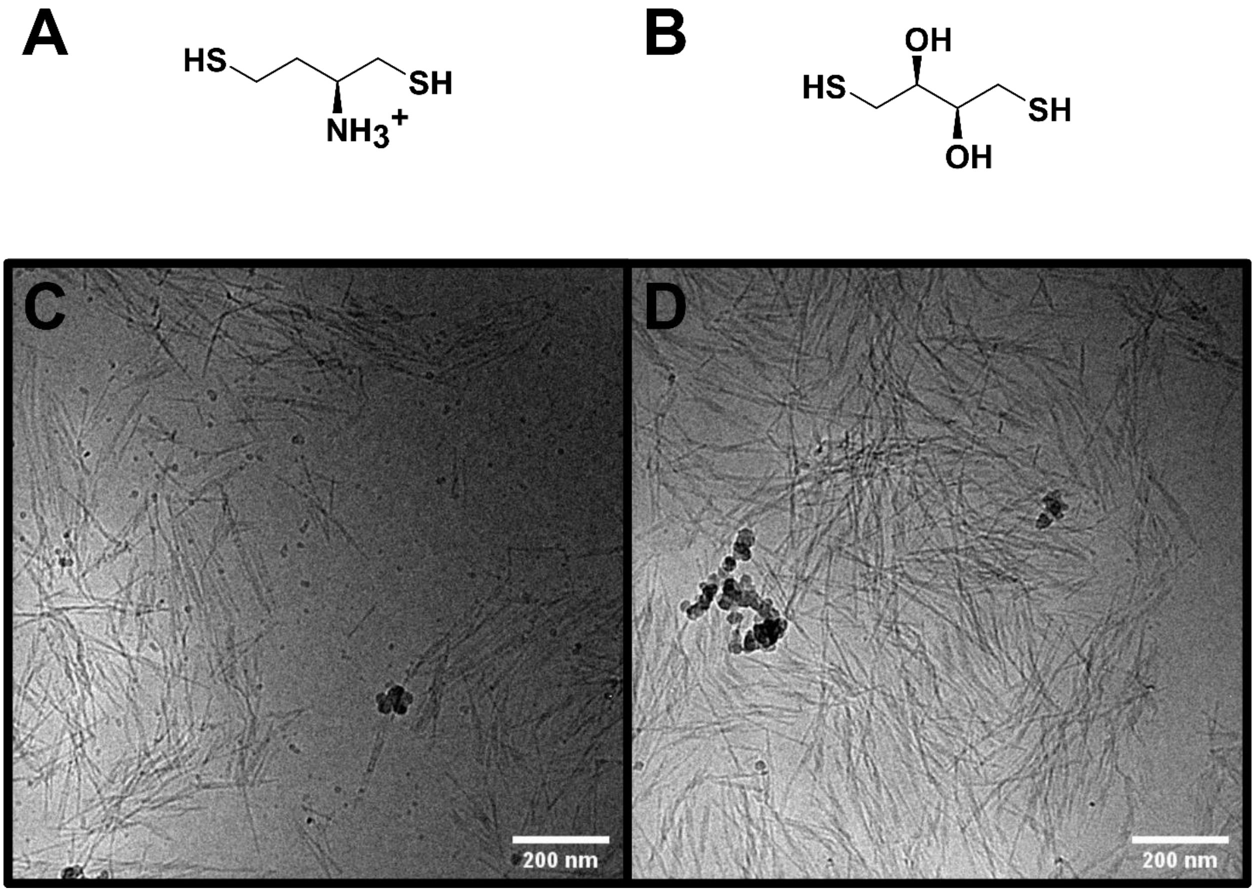

Figure S14: Dithiobutyl amine (DTBA) used in the synchronous process instead of DTT. A) Chemical structure of DTBA. B) Chemical structure of DTT for comparison C-D) CryoEM images of nanorods from a synchronous process using DTBA instead of DTT. These morphologies suggest some 2D stacking although it is not as prominent as some examples of the synchronous process with DTT (**S**).

Image Processing and Analysis

The stacked fiber phase observed in CryoTEM was analyzed with a custom cross-correlation based template matching algorithm (Figure S15). The stacked fiber phase has a periodic pattern of alternative dark fiber cores and bright Fresnel fringes. These periodic features are used to segment, or identify, the stacked fiber phase in each CryoTEM image collected in this study. Once the stacked fiber phase was segmented, the number of locally aligned fibers was labeled with the degree of stacking (DoS) (Figure 5). For example, if a region of the stacked fiber phase had 4 aligned fibers, it would be labeled with a degree of stacking value of 4. The resulting data were analyzed to track and quantify the stacked fiber phase across all timepoints in this study.

Stacked Fiber Phase Segmentation

Template Generation

Several symmetric templates of alternating black and white stripes were systematically generated to act as templates of the stacked fiber phase. Fibers of different thicknesses were accounted for by generating template sets with three different pixel spacings: 15, 20, and 25 pixels (Figure S16). Furthermore, templates were rotated 175 degrees at 5-degree intervals, resulting in 36 templates for each pixel spacing, and 108 templates in total. Finally, the templates were masked to be circular which makes them symmetric to all fiber directions, such that diagonal fibers do not have higher cross-correlations compared to vertical fibers.

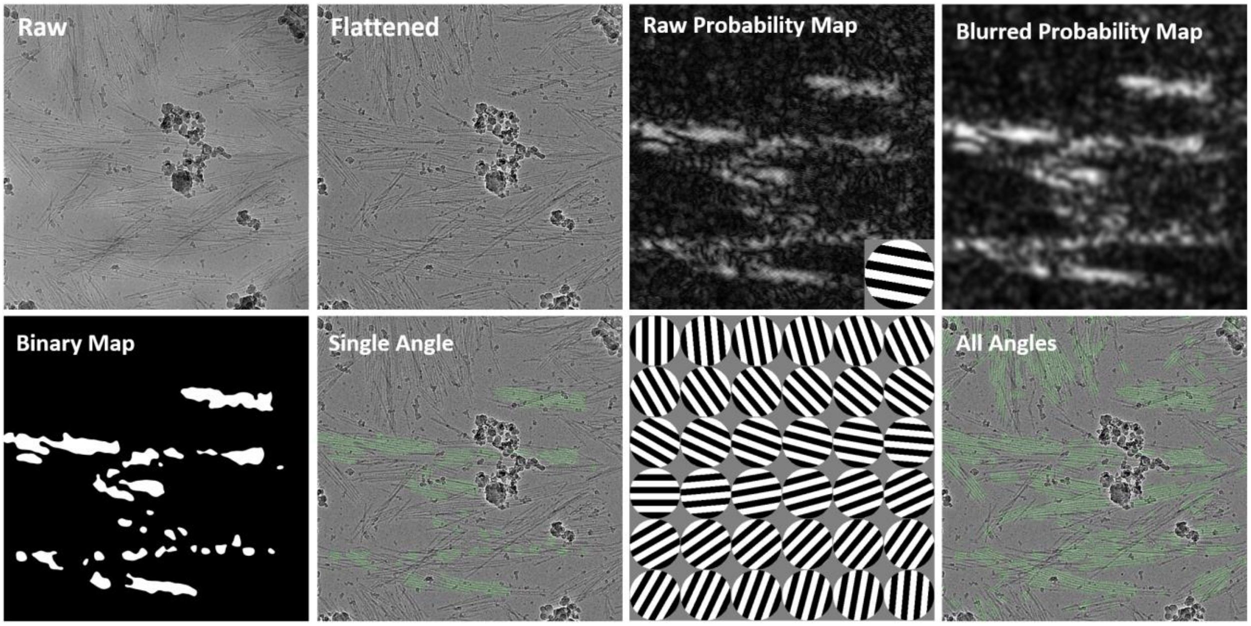

Figure S15: Image processing pipeline for stacked fiber phase segmentation. The image processing steps are outlined for a representative image. A more detailed description of each step can be found in the text below. The templates have been increased in size for display purposes.

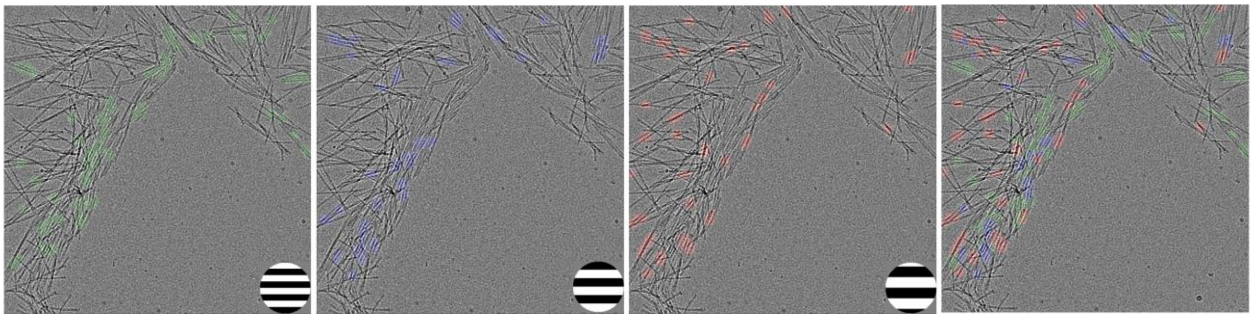

Figure S16. Individual segmentation maps for template pixel spacings of 15, 20, and 25 pixels. The templates have been increased in size by 4x for display purposes. The right figure shows an overlay of the 3 individual maps and demonstrates how different template spacings are used to identify different fiber stack spacings in the image. Note some highlighted regions are single fibers and not fiber stacks, these will be removed from the dataset during fiber stack labeling.

Stacked Fiber Phase Segmentation

Figure S15 shows the image processing pipeline applied to each CryoEM image. First, a high pass filter was applied to remove background features and create a flat image. Next, the normalized cross-correlation is computed between each template and the flattened image which results in a correlation probability map. The absolute value of this map is taken, which results in equal positive weight to correlation and anticorrelation. This is helpful because a high probability is returned both when the black pixels line up with the fibers and white pixels line up with the fringes (correlation), and also when white pixels line up with the fibers and black pixels line up with the fringes (anticorrelation). Next, the correlation probability map is smoothed with a Gaussian blur to smooth out the high correlation regions. Then, a probability threshold was used to convert the probability map into a binary segmentation map. Different probability thresholds were used for each image and were based on the estimated defocus of the image (Figure S17, details in 'Defocus Threshold Correction'). Binary maps for the 36 angles are calculated individually for each of the 3 templated spacings, resulting in 108 total binary maps. Finally, angles are combined across the 3 template spacings by adding the binary maps together for each angle, producing 36 binary maps which contain information about both the fiber stack location and fiber stack angle. Note the angular information is used to label the degree of stacking, as discussed in 'Labeling Degree of Stacking'.

Defocus Threshold Correction

It is well known that the brightness of the Fresnel fringes around an object will depend on the defocus of the microscope; larger microscope defocus values lead to higher intensity of the Fresnel fringes. We found that TEM images taken at higher defocus values were more sensitive to the segmentation algorithm and had higher normalized cross-correlations values. This makes physical sense, because the algorithm relies on the pattern of alternating dark fibers and bright fringes. If the fringes are brighter in some images due to differences in focus, it will increase the values of the normalized cross-correlations. To minimize this effect, all images were taken around 11 µm of defocus using the autofocus feature in SerialEM. However, there was still significant variation in defocus between images. To account for this variation in the segmentation algorithm, we estimated the defocus of every image and adjusted the probability threshold for segmentation on a per-image basis.

We estimated the defocus of every image by first calculating the Fast Fourier transform (FFT) image and then taking a radial integration (Figure S17A-B). Then, we used a peak-finding algorithm to find the distance to the second peak in the radial integration, which is characteristic of the defocus value (Figure S17C). The second peak was chosen because the first peak appeared distorted in many images, likely from high-frequency components generated by small features in the images such as fibers. We then took CryoEM images of amorphous ice with no sample at known

defocus values to use as a calibration curve to convert the second-peak distances to the defocus of the images (Figure S17D). Note that our algorithm was unable to detect peaks below 7 µm of defocus, but this was below the range of defocus values found in the experimental datasets. Once all images were labeled with the defocus value, the probability threshold for segmentation was adjusted with an empirically determined function (Figure S17E).

Originally, a static threshold of .145 was used to segment all images, which appeared to work well for most images. However, we observed that images taken at high defocus had inaccurate segmentation and were over segmented. We quantified this observation by examining the correlation between image defocus and the number of segmented pixels in an image, which should be random and uncorrelated features. To examine these features, we plotted the mean segmented pixels across images in different 1 µm defocus ranges, from 7-8 µm, 8-9 µm, 9-10 µm etc. (Figure S17F). Defocus and mean segmented pixels should be uncorrelated features and yet there is clearly a trend when a static threshold of .145 was used; high defocus images had a greater number of segmented pixels. After the defocus threshold correction was applied, the trend was reduced and a more random correlation between defocus and the number of segmented pixels was observed (Figure S17F). Figure S17G shows an uncorrected image which was over segmented while Figure S17H shows more accurate segmentation after the defocus threshold correction.

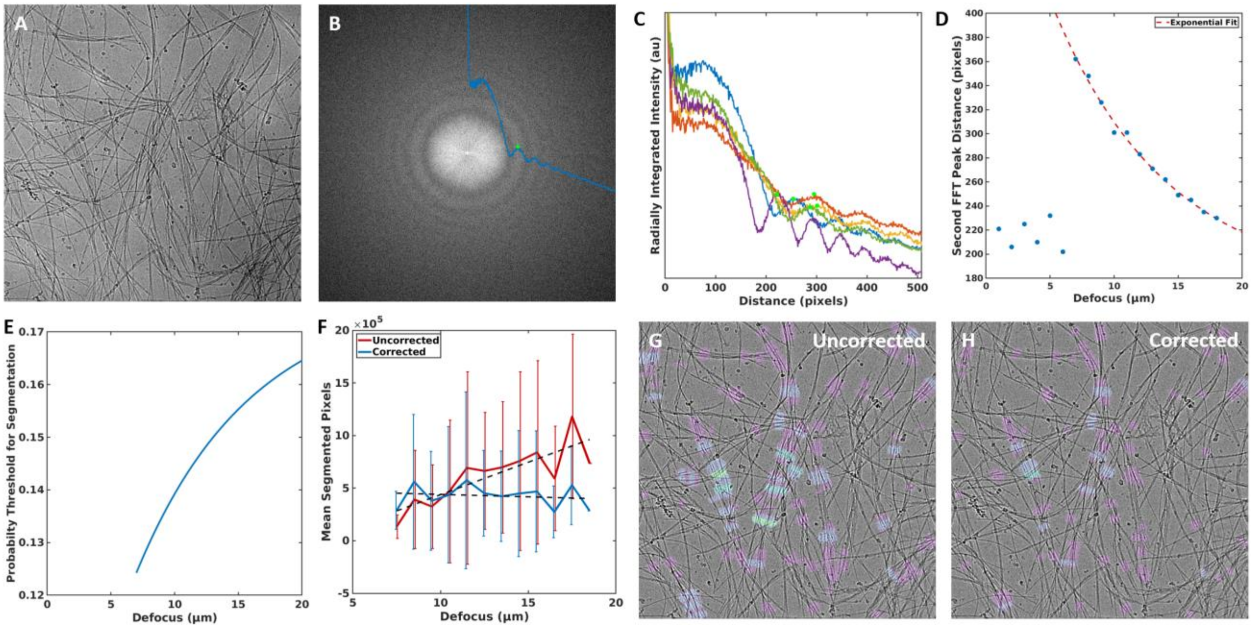

Figure S17. A) Typical TEM image with B) corresponding FFT image. Blue line represents radial integration of the FFT, with the green dot labeling the calculated second-peak distance. C) Examples of several radially integrated FFTs from images at different defocuses with the calculated second-peak distance. D) Calibration curve which relates defocus value to the calculated second-peak distance. Points below 7 µm were omitted from the fit. E) Empirically determined curve which adjusts probability threshold for segmentation as a function of image defocus. F) Mean segmented pixels of images across different defocus ranges. There was a clear correlation in the uncorrected curve which has been removed in the corrected curve. Dashed line represents linear fit. Error bars represent standard deviation. G) Uncorrected image with threshold of .145 showing over segmentation compared to H) segmentation after defocus threshold correction.

Labeling Degree of Stacking

After the stacked fiber phase was segmented, the goal was to track how well-ordered the stacked phase was by quantifying the degree of stacking (DoS) within each fiber stack structure. The DoS is the number of fibers present in the local region of the stacked fiber phase. Higher degrees of stacking are more ordered and entropically less

favorable than lower degrees of stacking. To understand how the structures were evolving during the sequential and synchronous processes, it was important to accurately quantify the amount and distribution of stacking present at each timepoint.

The output of the fiber stack phase segmentation algorithm is 36 binary maps of the stacked phase at each angle. Each angle was analyzed individually. For each binary object in the segmentation map, the stacked phase was cropped, then rotated to be oriented vertically. The rotation angle is equal to the rotation angle of the template. Once oriented vertically, a sliding window vertical integration was performed across 100 pixels (Figure S18A) to produce a high signal-to-noise 1D intensity profile (Figure S18B). A peak-finding algorithm was applied to find the intensity valleys created by the fibers, which were then labeled (Figure S18D). This resulted in a local label for the number of fibers at every location in the stacked phase. Small gaps in the labeling resulting from noise and non-fiber objects were smoothed over.

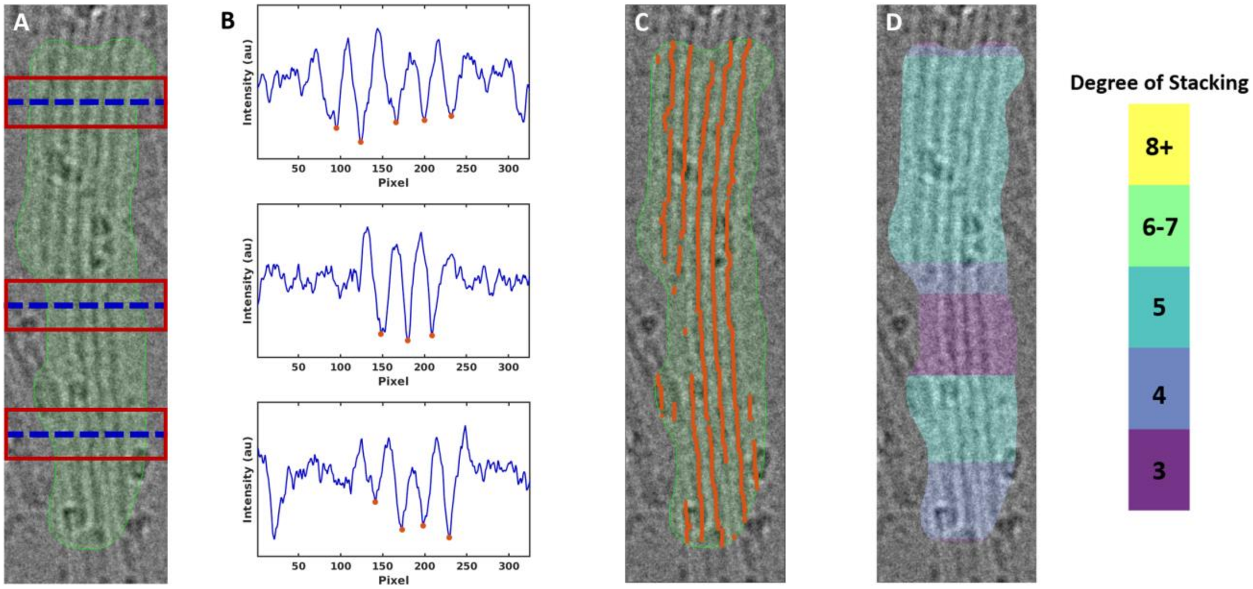

Figure S18. Labeling degree of stacking. A,B) snapshots of integrated profiles at different locations in the fiber stack. Fibers are labeled with orange dots. C,D) The number of fibers at each location is then counted and labeled.

Once each of the 36 angles were analyzed, they were combined into a single DoS map. Because of the large number of angles used, the same fiber stack was often segmented and labeled by 2 or more different angles of the templates. To resolve this conflict between angles and prevent multiple counting, the maximum DoS was taken for each labeled pixel. This was done because the most fibers were counted when the angle of the template best matched the angle of the stacked phase, and the highest DoS was recorded.

Fiber Stack Analysis

The stacked fiber phase segmentation and labeling image analysis pipeline was applied to 770 CryoEM images across 24 experimental timepoints, generating 398 million DoS datapoints. A datapoint is defined as a pixel labeled with the DoS. A subset of labeled images is shown in Figure S19. The data generated from segmentation and labeling was used to track the structure of the stacked fiber phase across all timepoints in this study.

We analyzed the mean DoS in each timepoint in order to clearly quantify the stacked phase of each process over time. The mean DoS presented in Figure 5E is determined by treating each image as an independent observation of the entire population of stacked fibers present within each timepoint. First, the “observed DoS” was calculated for each image by taking the sum of labeled DoS datapoints divided by the number of datapoints. By the central limit

theorem, the set of observed DoS values should have a Gaussian distribution even though the population of fiber stacks does not have a Gaussian distribution.

Next, the “mean observed DoS” was calculated for each timepoint, which is defined as the mean of the observations (Figure 5E). Importantly, this calculation gives each image (observation) an equal weighting in the calculation of mean observed DoS. This would assume each image contributes the same number of datapoints. However, due to the stochastic nature of the system, the distribution of datapoints is very heterogenous; some images contain a very small number of datapoints or no datapoints. To account for this and remove outliers, images contributing less than 2% of the total datapoints for a given timepoint were removed from the mean observed DoS calculation Next, we used the standard error and the t-distribution to compute the 95% confidence intervals of the mean, which are represented by the black error bars in Figure 5E and Figure S20. Accordingly, we are 95% confident the mean DoS of the entire population will fall within the error bars.

In addition to this analysis, the “mean combined DoS” was calculated for each timepoint (Figure 20). In this case, the datapoints from each image within a timepoint were combined into a single large dataset. Then, the mean DoS value of this combined dataset was calculated (mean combined DoS). In this calculation, each observation is not given an equal weighting in the mean; images with a large number of stacked fibers will contribute more heavily to the mean combined DoS. For comparison, the mean combined DoS was plotted with the mean observed DoS (Figure S20, green dots). Figure S20 shows the mean combined DoS values fall within the 95% confidence interval of the mean observed DoS values for all conditions.

Finally, we examined the distribution of DoS datapoints within each timepoint. Figure 5F shows a normalized bar graph of the distribution which clearly shows there is more high-order stacking in Sequential 1579 s, Synchronous 6060 s, and Synchronous 13440 s. Furthermore, we analyzed the amount of stacked phase present in the CryoTEM images (Figure S21). For each timepoint, the binary labeled pixels in each image were combined and then divided by the total pixels (image resolution multiplied by number of images in the timepoint). In addition, the percent coverage was calculated individually for each image within a timepoint and used to calculate the standard deviation of coverage. Note the standard deviation of labeled pixels treats all DoS values binary values.

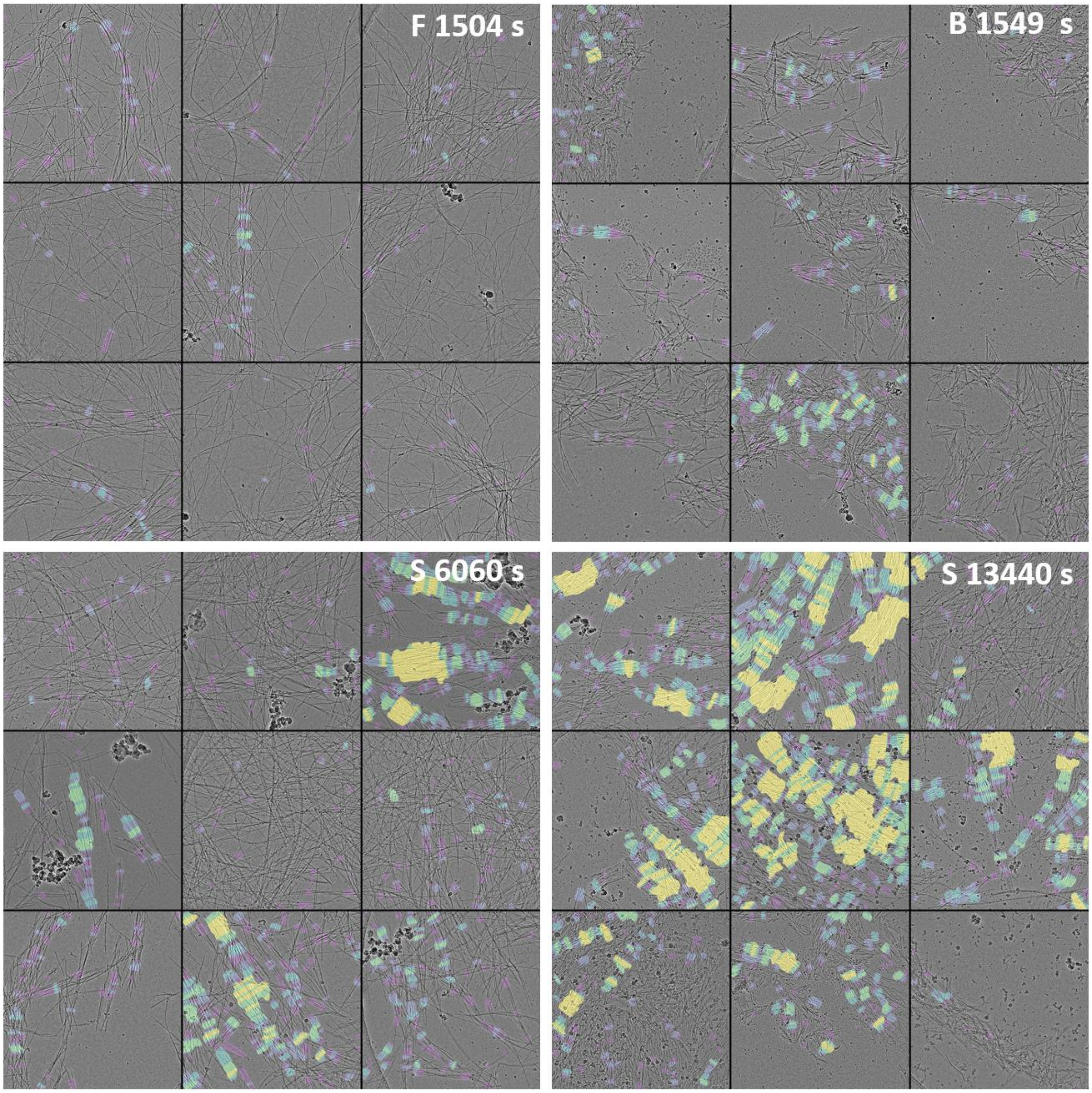

Figure S19. Examples of labeled images.

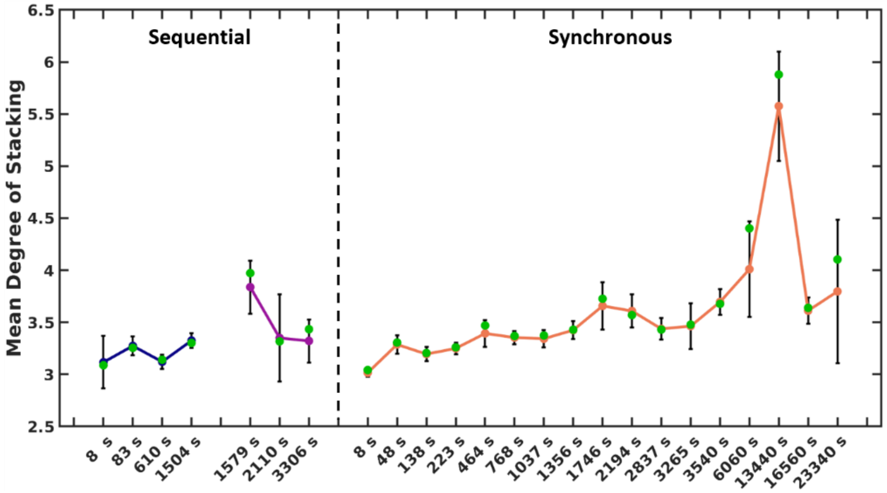

Figure S20. Display of Figure 5E with the mean combined DoS labeled for each timepoint (green dots).

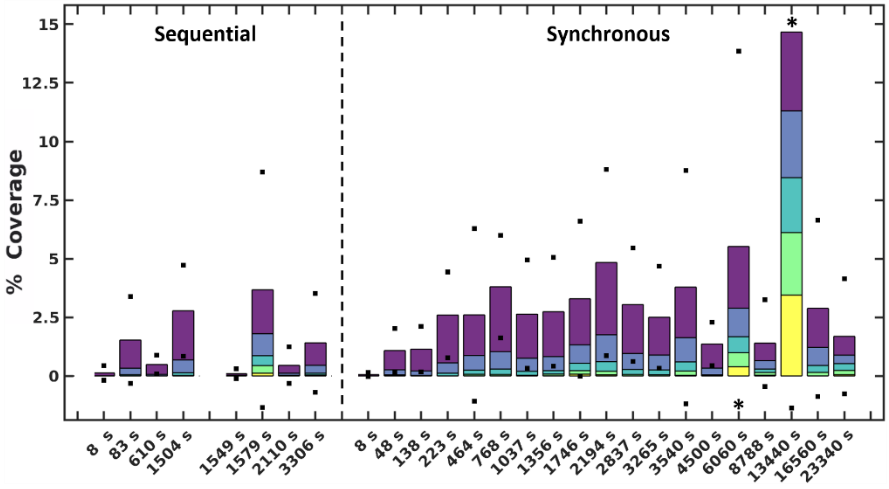

Figure S21. Density distribution of each timepoint, defined as the total number of labeled pixels divided by the total number of pixels in each timepoint. Black dots represent standard deviation of image densities within each timepoint. (*) represents standard deviation points omitted for visibility.

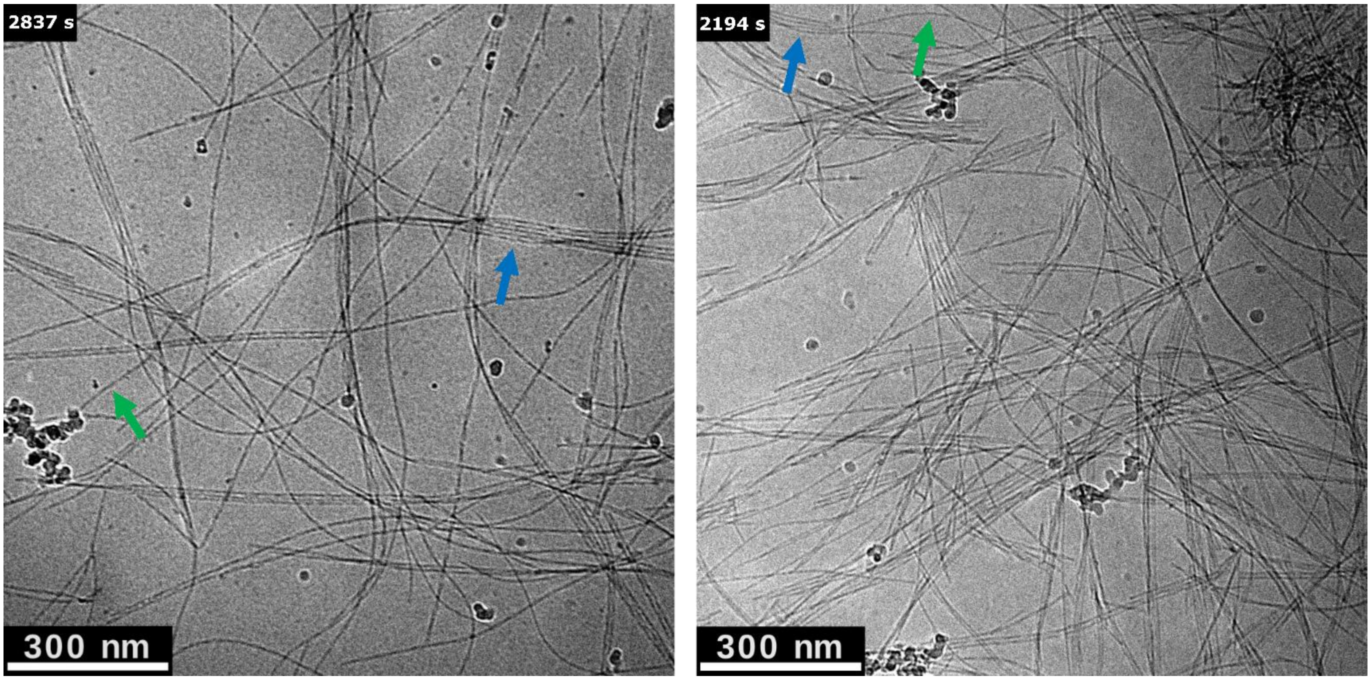

Figure S22. CryoEM images from the synchronous process (**S**) showing instances of fibers that are partially isolated and partially in a stack. Green arrows point to a segment of a fiber that is isolated whereas blue arrows point to a segment of a fiber that is participating in a stack.

Gillespie Simulations of Separable Self-assembly Process

Chemical mechanism

There are ten reactions in the chemical mechanism we consider here (Table S2). A distinguishing feature of the synchronous process is the abundance of fibers. To model this feature of the experiments, we add a reaction constituting fiber birth:

$$2\mathrm{CSSC_{sol}} \rightarrow 2\mathrm{CSSC_{fib}}$$

This reaction is necessary to generate information regarding the fibers formed and to ensure correct counting of CSSC species in solution and in fibers. Inclusion of a fiber birth reaction has been used previously to model the self-assembly of a similar fibrous material.[5]

For example, it includes the full balanced reactions accounting for the dissociation of CSH (R1) and DTT (R5). The main redox reactions involve the interconversion between dithiols and disulfide groups, CSH + CSH ⇌ CSSC + $2e^-$ + $2H^+$. The overall forward reaction 2CSH + H_2O_2 ⇌ CSSC + $2H_2O$ has the half reactions H_2O_2 + $2H^+$ + $2e^-$ ⇌ $2H_2O$ and 2CSH ⇌ CSSC + $2H^+$ + $2e^-$. The overall reverse reaction CSSC + DTT ⇌ 2CSH + DTT_{cyc} has the half reactions CSSC + $2H^+$ + $2e^-$ ⇌ 2CSH and DTT ⇌ DTT_{cyc} + $2H^+$ + $2e^-$.

Table S2. Chemical mechanism of CSSC/CSH system.

Number	Reaction	Forward rate constant	Reverse rate constant
1	$\mathrm{CSH + OH^- \rightleftharpoons CS^- + H_2O}$	$k_f^{\mathrm{CSH}} = 10^{pH-pK_a} k_r^{\mathrm{CSH}}$	$k_r^{\mathrm{CSH}} = rD(\mathrm{CSH})/V$
2	$\mathrm{CS^- + H_2O_2 \rightarrow CSOH + OH^-}$	$k_3 = 25\ M^{-1}s^{-1}$	
3	$\mathrm{CS^- + CSOH \rightarrow CSSC_{sol} + OH^-}$	$k_4 = 720\ M^{-1}s^{-1}$ source[6]	
4	$\mathrm{CSSC_{sol} \rightleftharpoons CSSC_{fib}}$	$k_7 = 1.46 * 10^{-17}\ M^{-1}s^{-1}$	$k_{sp} = 1000\ M^{-1}s^{-1}$
5	$\mathrm{DTT + OH^- \rightleftharpoons DTT^- + H_2O}$	$k_f^{\mathrm{DTT}} = 10^{pH-pK_a} k_r^{\mathrm{DTT}}$	$k_r^{DTT} = rD(\mathrm{DTT})/V$
6	$\mathrm{DTT^- + CSSC_{sol} \rightarrow DTTSC + CS^-}$	$k_6 = 5\ M^{-1}s^{-1}$	
7	$\mathrm{DTT^- + CSOH \rightarrow DTTSC + OH^-}$	$k_5 = 10\ M^{-1}s^{-1}$	
8	$\mathrm{DTTSC \rightarrow DTT_{cyc} + CSH}$	$k_d = 2.83 * 10^{-17}\ M^{-1}s^{-1}$	
9	$\mathrm{DTT^- + H_2O_2 \rightarrow DTTOH + OH^-}$	$k_1 = 0.0046\ M^{-1}s^{-1}$	
10	$\mathrm{DTTOH \rightarrow DTT_{cyc} + OH^-}$	$k_d = 2.83 * 10^{-17}\ M^{-1}s^{-1}$	
11	$\mathrm{2CSSC_{sol} \rightarrow 2CSSC_{fib}}$	$k_7 = 1.46 * 10^{-17}\ M^{-1}s^{-1}$	

Simulation Details

We simulate these chemical kinetics with the Gillespie Stochastic Simulation Algorithm (SSA)[7] with home-built code. The Gillespie SSA stochastically solves single realizations of the master equation, $\dot{p}(t) = Wp(t)$.[8] We simulate trajectories representing single evolutions of the species counts and fibers in time. The count of each species, fiber statistics, and reaction occurrence counts are recorded every $\Delta t = 0.1\ s$ for the forward, backward, and synchronous processes. The initial conditions for each simulations use the same concentrations of species and conditions as in the experiments within a simulation volume of $V = 5 * 10^{-18}\ L$. We take the rate constants of diffusion-limited reactions as the diffusion coefficient of the relevant species.[8]

We also generate statistics of fibers in these simulations. Each time the fiber birth reaction (Rxn. 11) is chosen, a new fiber is created that is two subunits in length. Once there is at least one fiber formed, reaction 4, the addition and removal of subunits to/from fibers via Rxn. 4 has a finite probability. Each time this reaction is selected

to occur, a fiber is chosen at random to gain or lose one subunit. If a fiber chosen to lose a subunit has a length of 2, that fiber is destroyed, and two subunits are released to the solution rather than one. At each interval on which the species counts are recorded ($\Delta t = 0.1\ s$), the number of fibers, mean length of fibers, and standard deviation of the length of fibers are also recorded.

The simulation code was written in C, and simulation output was analyzed in MatLab.

Rate constant determinations

To benchmark our simulations, we compare the profiles of various species counts and fiber statistics in time to experimental observations. The total time for each process to complete was the initial point of comparison. To match the timescales of simulations to experiments, we varied the rate constant values in the chemical mechanism. The goal of this was to find a single set of rate constant values that could be used in the forward, reverse, and synchronous processes that would match the timescale of experiments of the same processes. The forward process has the fewest unknown rate constants and was therefore considered first. The reverse process was subsequently analyzed while using the ranges of rate constant values determined in the exploration of the forward process. Following this, ranges of rate constant values were used from a combined understanding of the analysis of the forward and reverse processes to consider the synchronous process. The synchronous process was then optimized to be as close to the experimental timescale as possible by varying the remaining rate constants that were not important in the forward or reverse process or which had a range of possible values in the forward and reverse process.

In the forward process, only reactions 1-4 and reaction 11 occur. Of these reactions, only the rate constant for the dissociation of CSSC monomers from fibers (k_{sp}, reaction 4) is experimentally unknown. We varied the values of these rate constants in our simulations to match the simulation timescale of the three processes with the timescale of experiments. In addition to these, we varied the rate constant for reaction 2 (k_3) because we found in our simulations of the forward process that the experimentally determined value ($k_3 = 0.288\ M^{-1}s^{-1}$) did not result in any fiber growth. A series of pairs of values of k_3 and k_{sp} were identified for which the timescale of simulations closely matched the timescale of experiments. The timescale comparison was made by observing the profile of the count of subunits incorporated into fibers and the number of fibers in the simulations. These values plateaued around 1500 s, which matches the experiments whose fiber growth periods are largely, though not entirely, complete by 1500 s. We then used these pairs of values for the reverse process to narrow down the values of these rate constants to a single value each that can be held consistent over simulations of all three processes.

We then considered matching the timescale of the reverse process to experiments by using the narrowed scope of values for rate constants determined by optimization of the forward process. The reactions that occur in the reverse process are reactions 4-8. Of the rate constants corresponding to these reactions, the rate constants that do not appear to have been previously measured experimentally are k_{sp} and k_6. We determined a series of possible values of k_{sp} for the forward process. We therefore considered values of k_6 for each previously determined value of k_{sp}. In this optimization of the reverse process, we noted that an excess amount of H_2O_2 was used. Therefore, after the completion of the forward process, there is remaining H_2O_2 and CSOH in the solution. Reactions 9 and 10 then still need to be considered in the reverse process, though to a lesser extent than in the synchronous process. The profiles of the count of subunits incorporated into fibers and the count of fibers in time were again compared to experiments to determine the time at which the reverse process could be considered complete in our simulations. This time was then matched to experiments by varying the specified rate constant values.

In the synchronous process, all reactions in the chemical mechanism can occur. Of these reactions, we have a set of values of k_{sp}, k_3, and k_6 that, when used in our simulations, result in correct timescales of the forward and reverse processes from our previous analysis of these processes. The only remaining rate constant to determine is k_5, which corresponds to the deactivation of CSSC monomers in solution by DTT. We varied this rate constant for each set of values of the rate constants k_{sp}, k_3, and k_6, looking to match the timescale of simulations to the timescale of experiments. The best timing found through this method is one order of magnitude smaller than the timescale of experiments for the synchronous process but matches the timescale of experimental observations for the forward and reverse processes. The rate constant values determined from this analysis are provided in the table above.

Forward process

The forward process begins with equal concentrations of CSH and H_2O_2. The CSH species are quickly deprotonated to CS^-, Figure S23(a, gold). These resulting CS^- species then react with H_2O_2 to produce the alcohol CSOH, Figure S23(a, gray). The CSOH can then react with other CS^- to produce the subunit for assembly $CSSC_{sol}$ in solution. Subunits in solution assemble almost as quickly as they are formed, so only nominal amounts of free CSSC in solution are present in solution at any given time. Meanwhile, the length and number of fibers in solution grows quickly until there are no more available subunits to incorporate, Figure 23(b-c). The CS^- required for these reactions continue to be produced by deprotonation of CSH because the conjugate base CS^- is quickly consumed in subsequent reactions. Following full consumption of all CSH in solution, reactions adding and removing a subunit from the assembly dominates. Non-zero amounts of CSOH and H_2O_2 remain in solution at this point in time, Figure S23(a).

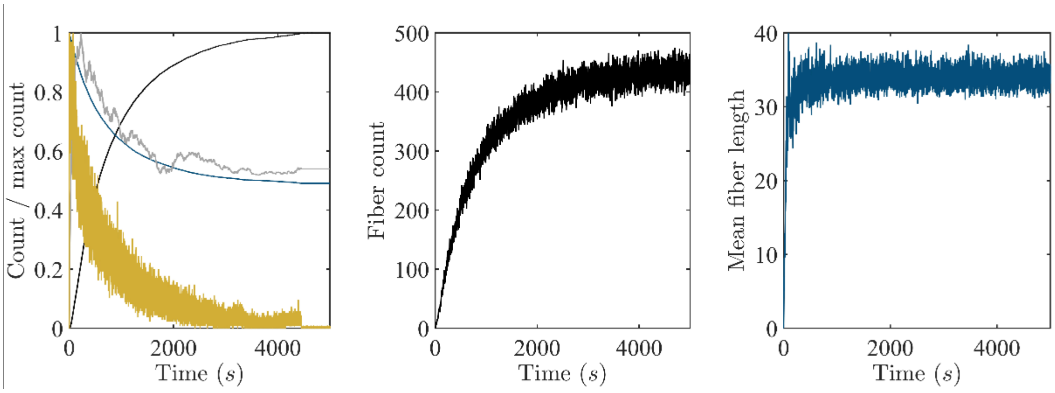

Figure S23. (a) Normalized count of assembled subunits $CSSC_{fib}$ (black), fuel H_2O_2 (blue), intermediate formative species CSOH (gray), and deprotonated CS^- (gold) in time reported in $\Delta t = 0.1\ s$ time intervals. **(b)** Count of fibers in time reported in $\Delta t = 0.1\ s$ time intervals. **(c)** Mean length of fibers in time reported in $\Delta t = 0.1\ s$ time intervals.

We can track the forward process not only with species counts (Figure S23(a)) and fiber statistics (Figure S 23(b-c)) but also through the count of occurrences of each possible reaction in the chemical mechanism. Initially, CSH is deprotonated (Figure S24(a, black)) in an effort to move toward an equilibrium concentration of CSH and its conjugate base CS^- as defined in the Henderson-Hasselbalch equation. However, the produced CS^- is quickly consumed by reaction with hydrogen peroxide in solution to produce CSOH and subsequent reaction of CSOH and CS^- to form subunits for assembly, Figure S24(b). Re-protonation of CS^- to CSH does occur (Figure S24(a, blue)), but the count of occurrences of this reaction are less than the count of occurrences of the deprotonation reaction because the product of deprotonation is used to make subunits in addition to being a reactant in the re-protonation reaction. Both of these reactions have a plateau in counts (Figure S24(a)) when all CSH is turned to CS^- and

subsequently used for subunit production. The subunits cannot be decomposed back to CS^- in the forward process due to the lack of DTT in solution. Therefore, with excess hydrogen peroxide in solution, all of the CSH initially in solution becomes either subunits CSSC or CSOH by the end of the forward process. This consumption of available CSH/CS^- is also the reason for the plateau in the count of occurrence of subunit-generating reactions.

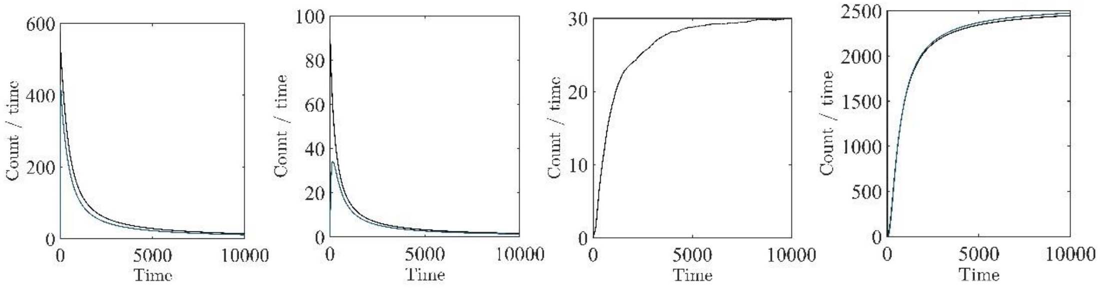

Figure S24. (a) Time profile of count of occurrences of reaction 1 per unit time, both forward (black) and reverse (blue) (the deprotonation of CSH and protonation of CS^-), reported in $\Delta t = 0.1\ s$ increments. **(b)** Time profile of count of occurrences of reactions 2 (black) and 3 (blue), formation of CSSC in solution, per unit time reported in $\Delta t = 0.1\ s$ increments. **(c)** Time profile of fiber births per unit time, reported in $\Delta t = 0.1\ s$ increments. **(d)** Time profile of count of occurrences of reaction 4, forward (black) and reverse (blue) (the addition and removal of subunits from fibers) per unit time, reported in $\Delta t = 0.1\ s$ increments.

The other three reactions (fiber birth, addition of CSSC to fiber, and removal of CSSC from fiber) that occur in the forward process do not exhibit plateaus in their count of occurrences when the forward process is complete at late times in simulations, Figure S24(c-d). This is because the addition and removal of CSSC to/from fibers continues after the depletion of CSH/CS^- in solution, Figure S24(d). Because these fluctuations between fibers and solution continue, enough CSSC (2 species in a simulation as the simulation is assumed to be well-mixed) can be present in solution at late times to continue to make new fibers and grow existing fibers.

Reverse process

Following completion of the forward assembly process, DTT can be added to solution to start the reverse (disassembly) process. DTT immediately begins to be deprotonated to DTT^-, Figure S27a, black). This deprotonated DTT can then scavenge any subunits in solution as well as any CSOH and H_2O_2 remaining in solution via reactions 6, 7, and 9. This scavenging process forms DTTSC and DTTOH. These species can then decompose via reactions 8 and 10, deactivating the contained DTT to DTT_{cyc} and preventing that resulting molecule of DTT_{cyc} from having subsequent reactions. DTT_{cyc} in solution increases over time as a result, Figure S25(a, blue). Through this process, the DTT in solution decreases quickly while fibers are disassembled, Figure S25(b-c). The amount of DTT^- in solution plateaus after approximately 2 minutes at a non-zero value. At this time, the number of fibers in solutions plateaus at a low value, Figure S25(b). This corresponds to experiments, where all or nearly all of the gel is consumed by the end of the reverse process.

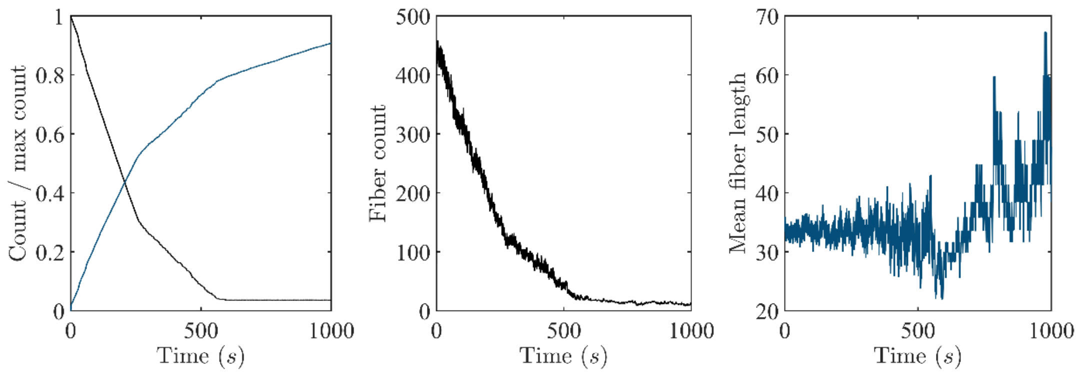

FigureS25. (a) Normalized count of assembled subunits $CSSC_{fib}$ (black) and deactivated scavenging species DTT_{cyc} (blue) in time reported in $\Delta t = 0.1\ s$ time intervals. **(b)** Count of fibers in time reported in $\Delta t = 0.1\ s$ time intervals. **(c)** Mean length of fibers in time reported in $\Delta t = 0.1\ s$ time intervals.

The speed at which fibers are disassembled is controlled by how quickly subunits detach from fibers (through the rate constant k_{sp}) and how quickly DTT^- can scavenge those subunits in solution (through the rate constant k_6). Both rate constants must be sufficiently large for fibers to disassembled and the reverse process to go forward. Both rate constants are determined through the means specified before.

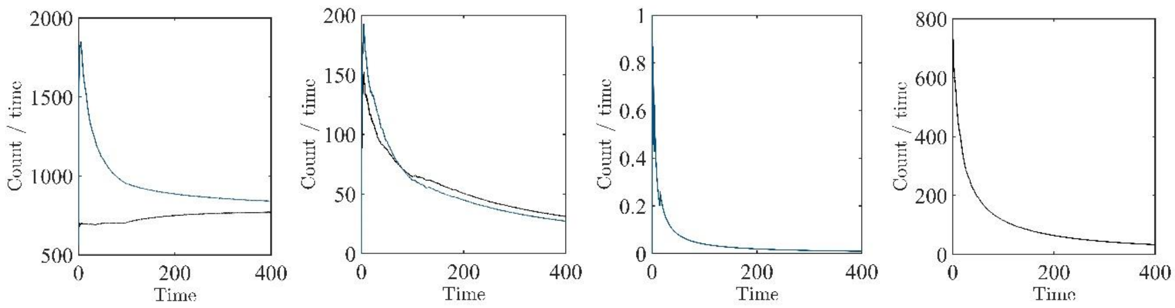

FigureS26. (a) Time series of count of occurrences of CSH deprotonation (black) and CS^- protonation (blue) per unit time, reported in $\Delta t = 0.1\ s$ increments. **(b)** Time series of count of occurrences of reaction 2 (black) and 3 (blue) to form CSSC per unit time, reported in $\Delta t = 0.1\ s$ increments. **(c)** Time series of count of occurrences of fiber birth (black, zero for all time) and CSSC addition to fibers (blue) per unit time, reported in $\Delta t = 0.1\ s$ increments.

(d) Time series of count of occurrences of CSSC removal from fibers per unit time, reported in $\Delta t = 0.1\,s$ increments.

We can also track the count of occurrences of each reaction that is possible in the reverse process. Following introduction of DTT into solution, DTT is rapidly deprotonated, Figure S27(a, black). At the same time, subunits CSSC are incorporated into fibers and unincorporated from fibers, Figure S26(c-d). The DTT^- resulting from deprotonation of DTT in solution can then scavenge excess H_2O_2 remaining in solution from the forward process via reaction 9, CSOH remaining in solution (and, to a lesser extent, CSOH formed during the decay of fibers by excess hydrogen peroxide from the forward process) via reaction 7, and CSSC in solution via reaction 6 as subunits incorporate and unincorporate (reaction 4 forward and reverse) from fibers. Of these, CSSC is scavenged the most and hydrogen peroxide the least, Figure S27(b). The scavenging of hydrogen peroxide has a slower rate constant than hydrogen peroxide reacting with CS^- formed in the decomposition of fibers. This scavenging reaction therefore occurs the least. This process rapidly decays the fibers. At the same time, a small amount of fiber growth reactions occur (Figure S26(c, blue)), though no new fibers are formed, Figure S26(c, black).

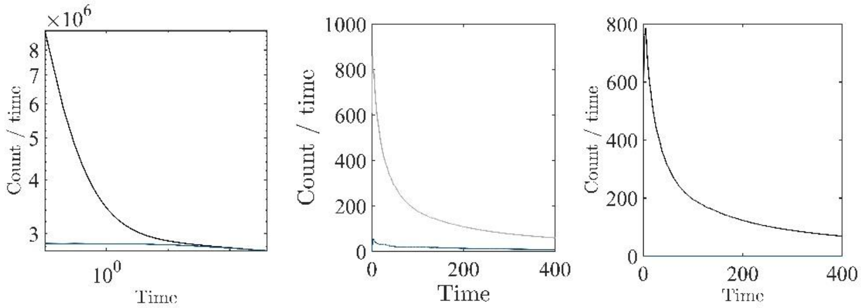

FigureS27. (a) Time series of count of occurrences per unit time of DTT deprotonation (black) and protonation of DTT^- (blue), lines overlap, reported in $\Delta t = 0.1\,s$ increments. **(b)** Times series of count of occurrences per unit time of DTT^- scavenging of hydrogen peroxide (black, zero for all time), CSOH (blue), and CSSC in solution (gray), reported in $\Delta t = 0.1\,s$ increments. **(c)** Time series of count of occurrences per unit time of decomposition of DTTSC (black) and DTTOH (blue, zero for all time), reported in $\Delta t = 0.1\,s$ increments.

Synchronous process

In the synchronous process, CSH, DTT, and H_2O_2 are initially all present in solution. Following the initial formation of subunits and fibers, the reactions associated with both the forward and reverse process can then proceed simultaneously. This process increases the number of fibers in solution and the length of those fibers for an extended time (until approx. 3000 s), Figure S28(b-c). During this time, fibers are being created and lengthened. While the fiber decay process does occur during this time, the consumption of hydrogen peroxide fuels fiber formation and growth. There are fluctuations in the number of fibers and the mean length of fibers associated with the simultaneous decay process. However, the overall trend we see during this time is fiber birth and growth.

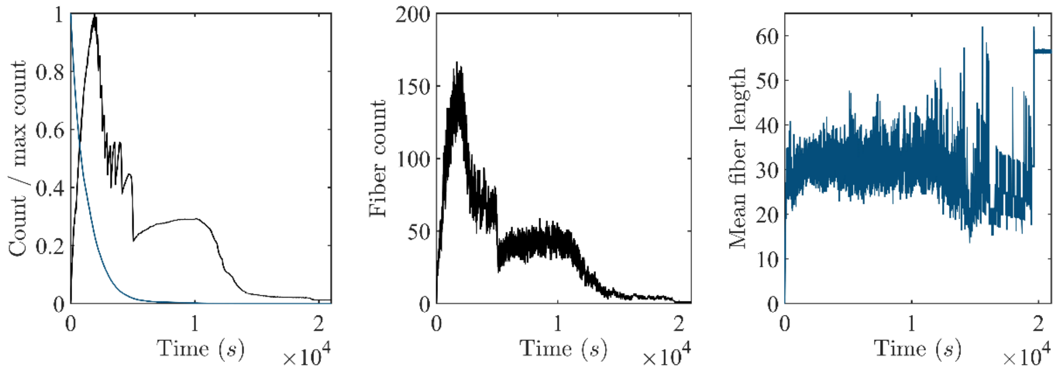

FigureS28. (a) Normalized count of assembled subunits $CSSC_{fib}$ (black) and fuel H_2O_2 (blue) in time reported in $\Delta t = 0.1\ s$ time intervals. Dashed vertical line indicates time of peak amount of CSSC incorporated into fibers. **(b)** Count of fibers in time reported in $\Delta t = 0.1\ s$ time intervals. **(c)** Mean length of fibers in time reported in $\Delta t = 0.1\ s$ time intervals.

After approximately 3000 s, the disassembly process dominates, Figure S S28(a-c). This occurs when the H_2O_2 in solution is largely depleted, Figure S28(a, blue). However, the maximum in the fiber count as well as the maximum in the mean length of fibers occur before the hydrogen peroxide is fully consumed as the decreasing concentration of hydrogen peroxide at this time decreases the propensity of its reaction with CS^-, Figure S28(a-b).

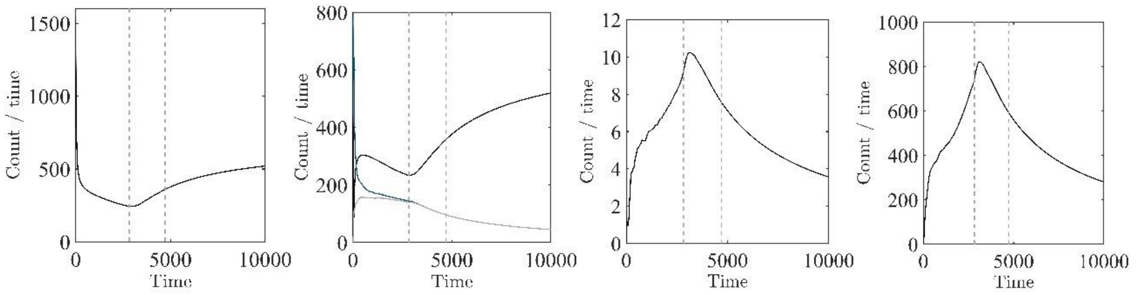

FigureS29. (a) Time series of count of occurrences per unit time of CSH deprotonation reported in $\Delta t = 0.1\ s$ time intervals. **(b)** Time series of count of occurrences per unit time of CS^- protonation (black), CSOH formation (blue), and CSSC formation (gray) reported in $\Delta t = 0.1\ s$ time intervals. **(c)** Time series of count of occurrences per unit time of fiber births (black) reported in $\Delta t = 0.1\ s$ time intervals. **(d)** Time series of count of occurrences per unit time of subunit incorporation into fibers reported in $\Delta t = 0.1\ s$ time intervals.

The full consumption of H_2O_2 in solution (due to reaction with CS^- and DTT^-) prevents subunits CSSC to be reformed and reincorporated into fibers. This occurs near 3000 s in these data, as indicated by the plateau in the occurrence of the reaction for the formation of CSSC (Figure S29(b, gray)), the maximum in the number of subunits incorporated into fibers (Figure S28(a, black)), and the slowing to a plateau of the reaction incorporating subunits into fibers (Figure S29(d)). This essentially stops the reassembly process and therefore the transient assembly as there is no more fuel to dissipate to perpetuate the process.

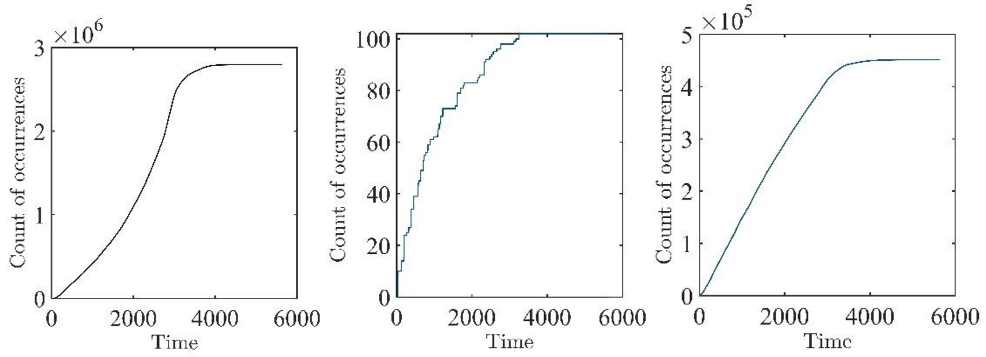

FigureS30. (a) Time series of count of occurrences of DTT deprotonation reaction reported in $\Delta t = 0.1\ s$ time intervals. **(b)** Time series of count of occurrences of scavenging reactions of CSOH (black, nearly zero) and CSSC (blue) by DTT^- reported in $\Delta t = 0.1\ s$ time intervals. **(c)** Time series of count of occurrences of decomposition of DTTOH (black, nearly zero) and DTTSC (blue) reported in $\Delta t = 0.1\ s$ time intervals.

Following this time when the hydrogen peroxide fuel in solution is fully consumed, fibers are rapidly degraded. The scavenging reactions of DTT^- and subsequent decomposition of the product of scavenging continue until about 5000 s (Figure S30(b-c)), by which point all or most of the CSSC, CSOH, and H_2O_2 in solution are consumed (Figure S28(a)). As an excess amount of DTT is provided in solution, the remaining DTT and DTT^- in solution quickly reach their equilibrium concentrations. As the remaining CSSC and CSOH are being degraded, CSH and CS^- also go to their equilibrium concentrations.

A phase of short fibers stacking in parallel is observed in experiments for a (relatively) extended period of time in the synchronous process, for a brief period of time in the reverse process, and not at all in the forward process. We surmise this is because fibers have time to grow to greater lengths and diffuse away from each other without also being simultaneously decayed when the process is carried out sequentially. The diffusion of fibers away from each other prevents there from being dense pockets of decaying fibers, as occurs in the synchronous process. There is then less entropic driving toward creating the stacked conformation in the sequential process. This can happen because fibers are not being decayed while they are simultaneously being built in the forward process. Allowing both of these processes to happen simultaneously (i.e., the synchronous process) prevents fibers from growing as long as they otherwise would in the sequential process. This decay in the synchronous process is also happening when fibers would otherwise be diffusing away from each other with very little decomposition in the sequential process. The occurrence of this stacked phase in the reverse process is also likely prolonged in the synchronous process due to the general lengthening of the timescale on which the synchronous process occurs. This lengthening is a direct result of the building and decaying of fibers being carried out simultaneously.

Cycle

From our simulations, the defining feature that distinguishes the synchronous process from the sequential process is a set of reactions that together constitute a “dissipative” cycle. This is a subset of reactions that leads to the repeated growth and decay of fibers. These reactions that form the cycle can be separated into those that lead to the growth of fibers and those that lead to the decay of fibers. The fiber growth reactions in this cycle are reactions 2 and 3, which together form the subunits for assembly CSSC. These reactions occur in all three processes. However, these reactions occur much less frequently in the reverse process than the forward process and only as a result of H_2O_2 and CSOH remaining in solution from the forward process. The fiber decay reactions in this cycle are reactions 6 and 8, which involve the scavenging of free subunits CSSC by DTT and subsequent decay of the resulting DTTSC. These reactions are not possible in the forward process due to the lack of DTT in solution but occur in both

the reverse and synchronous processes. Further, the decomposition of DTTSC produces CS^-, a reactant in both reactions 2 and 3, which form the other half of the cycle.

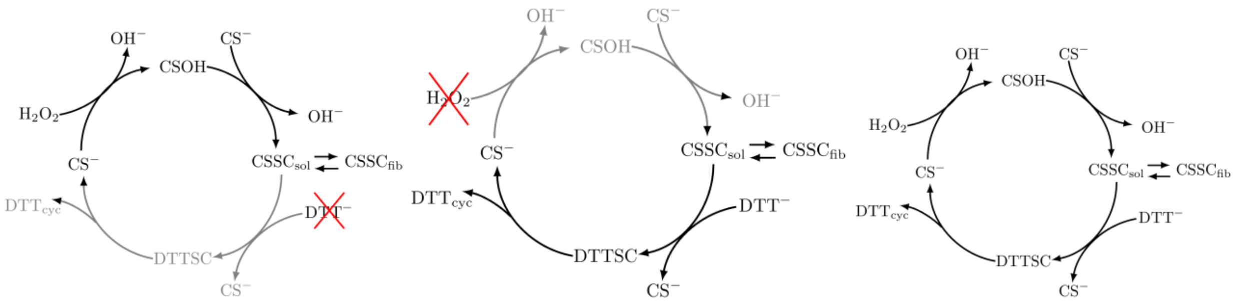

Figure S31. (a)

To quantitatively measure the difference between the synchronous and sequential processes, we calculated the number of times the system goes through this cycle. We measured this here via the smallest count of reaction occurrences of the four reactions identified in the cycle—and only for simulations using our optimized rate constants. In the forward process, the system cannot undergo this cycle because half of the reactions constituting the cycle lack reactants. In the reverse process, this cycle can occur but only due to residual H_2O_2 and CSOH in the system. A limited number of cycle traversals are possible as a result (8,776 on average over 10 simulations). By comparison, the "dissipative" cycle has sufficient reactants to freely traverse this cycle many times (90,317 on average over 10 simulations). This prolongs the dis/assembly process by allowing both subunit production and scavenging to occur simultaneously. This cycle continues to be driven by the consumption of chemical fuel: hydrogen peroxide in solution, which is necessary to produce subunits.

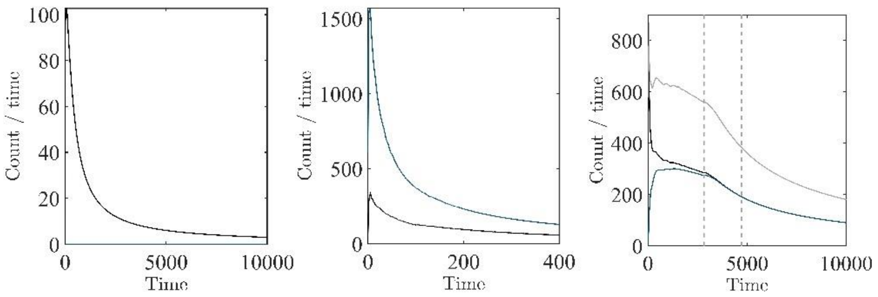

FigureS32. (a) Sum of occurrences per unit time of reactions 2 and 3 (forward/production half of cycle) vs time reported in $\Delta t = 0.1\ s$ intervals for the forward process. No reactions for the reverse/destruction half of the cycle occur. **(b)** Sum of occurrences per unit time of reactions 2 and 3 (black) and reactions 6 and 8 (blue) vs time reported in $\Delta t = 0.1\ s$ intervals for the reverse process. **(c)** Sum of occurrences per unit time of reaction 2 and 3 (black), reactions 6 and 8 (blue), and reactions 2, 3, 6, and 8 (gray) vs time reported in $\Delta t = 0.1\ s$ intervals for the synchronous process with vertical lines marking the time of maximal number of fibers and time of final plateau.

The production half of this cycle occurs in all three processes but to differing amounts, Figure S32(a-c). The reverse process has about 63% of the reaction occurrences of reactions 2 and 3 compared to the forward process, Figure S32(b). This decrease is a result of only residual amounts of hydrogen peroxide and CSOH in solution from the forward process. Conversely, the synchronous process goes through 309% the occurrences of reactions 2 and 3 that the forward process does, Figure S32(c). This is possible through the regeneration of CS^- through this cycle allowing new subunits to be formed until the hydrogen peroxide fuel is expended.

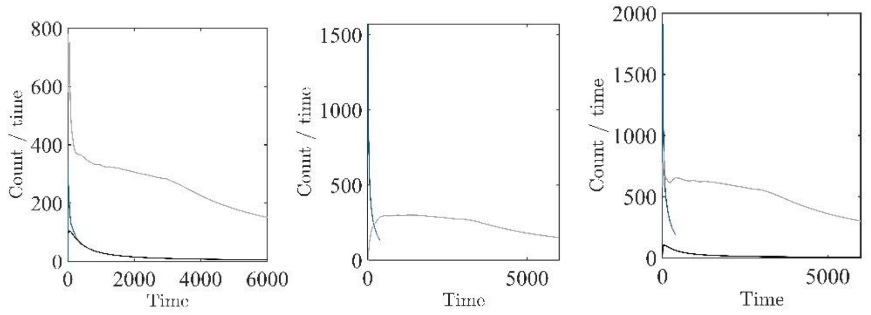

FigureS33. (a) Sum of occurrences per unit time of reactions 2 and 3 (forward/production half of cycle) vs time reported in $\Delta t = 0.1\ s$ intervals for forward process (black), reverse process (blue), and synchronous process (gray). **(b)** Sum of occurrences per unit time of reactions 6 and 8 (reverse/destruction half of cycle) vs time reported in $\Delta t = 0.1\ s$ intervals for forward process (black, zero for all time), reverse process (blue), and synchronous process (gray). **(c)** Sum of occurrences per unit time of reactions 2, 3, 6, and 8 (all of cycle) vs time reported in $\Delta t = 0.1\ s$ intervals for forward process (black), reverse process (blue), and synchronous process (gray).

The destruction half of this cycle occurs only in the reverse and synchronous processes. The reverse process undergoes these reactions and quickly decomposes the entire assembly. However, the synchronous system can regenerate reactants for the reproduction of subunits for assembly. As a result, the synchronous process has 309% the occurrence of reactions 6 and 8 that the reverse process has, Figure S33(b-c).

Varying initial amount of hydrogen peroxide in the synchronous process

Given that hydrogen peroxide acts as a fuel for the "dissipative" cycle that occurs in the chemically-driven synchronous process, we hypothesize that the initial concentration of hydrogen peroxide will have some effect on the timing and yield of the fibrous material. To analyze this effect, we varied the initial concentration of hydrogen peroxide provided for the synchronous process with all other initial conditions the same as previously. We then observed several important points of time in the simulation data that mark changes in the behavior of the synchronous process: the positions in time and value of the maximal amount of CSSC incorporated into fibers, the maximal number of fibers, the start of the plateau in the number of CSSC incorporated into fibers at long times, the start of the plateau in the number of fibers at long times, the time at which the fuel is no longer consumed linearly in time, and the time at which the amount of fuel is so small in simulations as to be considered depleted.

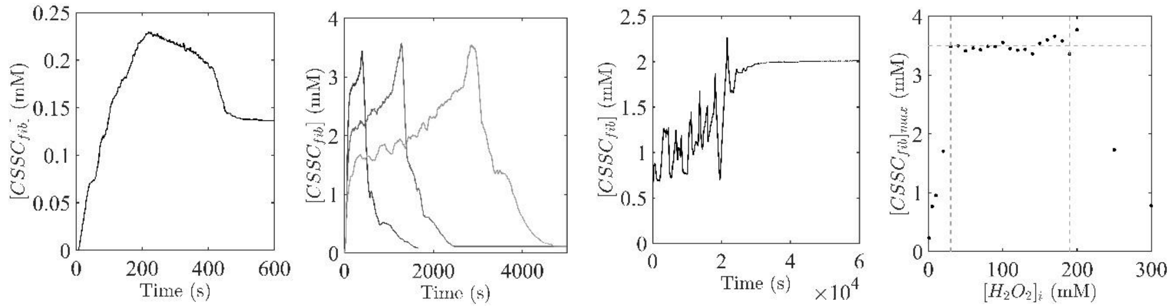

FigureS34. (a) Concentration of subunits in fibers $[CSSC_{fib}]$ (mM) vs time (s) for starting concentration of hydrogen peroxide of $[H_2O_2]_i = 1\ mM$. **(b)** Concentration of subunits in fibers $[CSSC_{fib}]$ (mM) vs time (s) for starting concentration of hydrogen peroxide of $[H_2O_2]_i = 50, 100, 150\ mM$ (lighter color is increased concentration). **(c)** Concentration of subunits in fibers $[CSSC_{fib}]$ (mM) vs time (s) for starting concentration of hydrogen peroxide of $[H_2O_2]_i = 240\ mM$. **(d)** Maximal concentration of subunits in fibers (mM) vs initial concentration of hydrogen

peroxide (mM). Vertical lines denote minimal and maximal initial concentrations for synchronous process to proceed as previously detailed. Horizontal line denotes mean maximal concentration of subunits in fibers.

When we varied the initial amount of hydrogen peroxide supplied to the system between 0.1 mM and 1000 mM, we found that the maximal number of subunits incorporated into fibers, the number of fibers, the final plateau value in the amount of subunits incorporated into fibers, and the final plateau in the amount of fibers did not vary significantly with initial concentrations of hydrogen peroxide between 30 mM and 200 mM, Figures. 34(d), 33(a-b). Below this range, the amount of fuel provided was insufficient to sustain the transient assembly for a prolonged time. Above this range, the among of fuel provided is enough to ensure that the total yield of subunits in fibers is maximal (all possible subunits are incorporated into fibers) and sustained. At such high concentrations of the fuel, fibers are not decayed to appreciable amounts, and the species profiles and fiber statistics resemble those of the forward process.

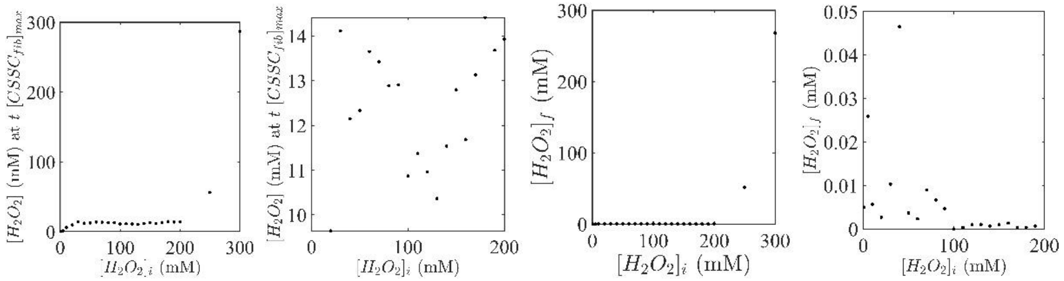

Figure S35. (a) Concentration of hydrogen peroxide at the time of the maximal concentration of subunits in fibers vs. initial concentration of hydrogen peroxide. **(b)** Concentration of hydrogen peroxide at the time of the maximal concentration of subunits in fibers vs. initial concentration of hydrogen peroxide only within range of initial concentrations of hydrogen peroxide resulting in normal synchronous process. **(c)** Final concentration of hydrogen peroxide vs. initial concentration of hydrogen peroxide. **(d)** Final concentration of hydrogen peroxide vs. initial concentration of hydrogen peroxide only within range of initial concentrations of hydrogen peroxide resulting in normal synchronous process.

We also found that within the range of 30 mM to 200 mM of hydrogen peroxide initially provided, the amount of hydrogen peroxide remaining at the time when the number of subunits incorporated into fibers is maximal also does not vary appreciably, Figure S35(c-d). This finding suggests that for a given initial concentration of CSH and DTT, there is a threshold concentration of hydrogen peroxide in solution above which the process switches from being primarily characterized by fiber formation and growth to being primarily characterized by fiber decay and death.

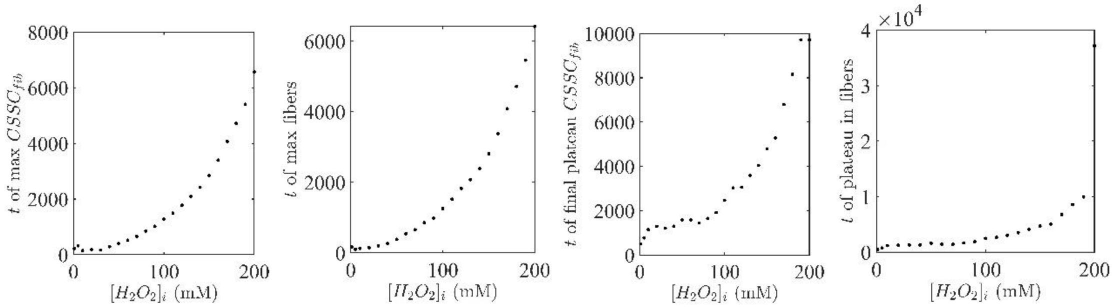

Figure S36. (a) Time (s) of maximal subunits in fibers vs. initial concentration of hydrogen peroxide (mM). **(b)** Time (s) of maximal number of fibers vs. initial concentration of hydrogen peroxide (mM). **(c)** Time (s) of final plateau in number of subunits in fibers vs. initial concentration of hydrogen peroxide (mM). **(d)** Time (s) of final plateau in number of fibers vs. initial concentration of hydrogen peroxide (mM).

While the counts of species/fibers did not vary in a systematic way with the controlled variation in the initial concentration of hydrogen peroxide, the times at which these important changes in trends occurred did vary in a systematic fashion, Figure S36. The time of maximal subunits in fibers and the time of maximal count of fibers are almost identical, Figure S36(a-b). Both times vary systematically with the initial concentration of hydrogen peroxide in solution approximately via the equation $t\ (s) = 130 * \exp(0.0212 * [H_2O_2]_i)$. The other two pertinent times do not vary with as clean a trend, Figure S35(c-d). We conclude from these data that the time to reach the peak number of subunits in fibers is a nonlinearly increasing function of the initial concentration of hydrogen peroxide.

Maintain fiber count by injection of hydrogen peroxide

From our simulations, a plausible design principle emerged: because of the nonlinear nature of the system, smaller amounts of fuel might be necessary to sustain the fibers compared to the amount of fuel needed to create them initially. To test this hypothesis, we added small doses of fuel in the synchronous process to sustain the fiber population when the fiber population was nearly extinct. Using the simulation setup above, we attempted to maintain a specified count of fibers in the simulation by setting a desired threshold count of fibers below which a specified amount of hydrogen peroxide would be injected into the system. In these simulations, it was also important to set a limit on the frequency with which hydrogen peroxide can be injected into the system. If the frequency is too high, there is an abundance of hydrogen peroxide in solution rather than doping the system with small quantities of hydrogen peroxide.

With this constraint, the system proceeds as normal for the synchronous process until fibers begin to collapse, Figure S37(a). In the time where fiber collapse dominates, injection of hydrogen peroxide proceeds when sufficient fibers have decayed to cross the threshold value. At this point, hydrogen peroxide is injected into the system. Each time hydrogen peroxide is injected, fibers grow for a short period of time before fibers collapse down to the threshold again. The system then oscillates between fiber growth just after an injection of hydrogen peroxide and fiber collapse following the depletion of that injected amount of hydrogen peroxide. These oscillations continue for an extended period of time (over 15,000 s). The amount of hydrogen peroxide injected into the system at any one time is significantly smaller than the initial amount of hydrogen peroxide in the system, Figure S37(b, red).

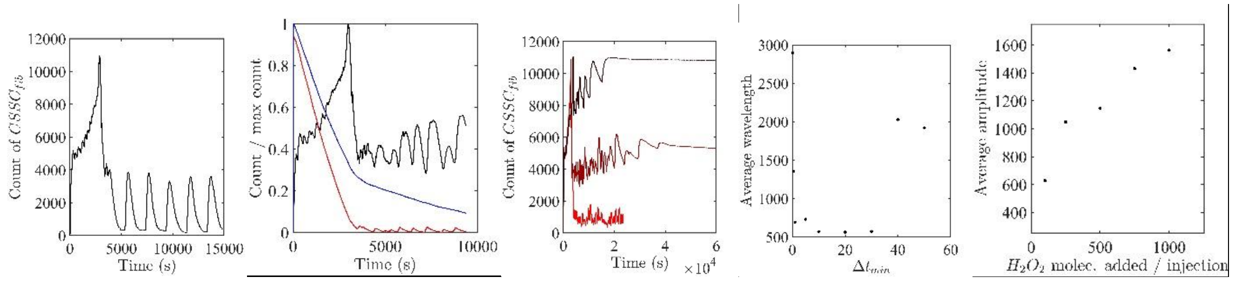

Figure S37. (a) Count of subunits in fibers while injecting 500 molecules of hydrogen peroxide no more often than every 2,000 s if the number of fibers falls below 50. **(b)** Normalized time profile of count of subunits in fibers (black), hydrogen peroxide (red), and deprotonated DTT (blue) recorded in $\Delta t = 0.1\ s$ increments with a threshold of 50 fibers and injecting 500 molecules of hydrogen peroxide no more often than every 5 s. **(c)** Count of subunits in fibers while injecting 500 molecules of hydrogen peroxide no more than every 10 s if the number of fibers falls below 10 (bright red), 50 (dark red), and 100 (black). **(d)** Average wavelength of oscillations in subunits in fibers time series at late times for a given allowed frequency of injection $\Delta t \in \{0.1\ s, 0.5\ s, 1\ s, 5\ s, 10\ s, 20\ s, 30\ s, 40\ s, 50\ s\}$ for threshold number of fibers 50 and 500 molecules of hydrogen peroxide added per injection. **(e)** Average amplitude of oscillations in subunits in fibers time series at late times for a given number of molecules to add per injection (100, 250, 500, 750, 1000) for threshold number of fibers 50 and an allowed frequency of injection of $\Delta t = 10\ s$.

In this process, the frequency at which injections are allowed, the amount of hydrogen peroxide to inject at a time, and the threshold number of fibers below which to inject hydrogen peroxide can all be varied. We now explore each of these.

Frequency of injection. One might expect the frequency of injection to be closely related to the wavelength of oscillations. However, the wavelength of these oscillations is not the same as the allowed frequency of injection, Figure S37(d). The period of time for a single wave of this oscillation is longer than the allowed frequency of injection of hydrogen peroxide. This suggests that the wavelength of these oscillations is indicative of the amount of time the system can use a given quantity of hydrogen peroxide to sustain fiber growth. The allowed frequency of injection can be small enough that the system becomes flooded with fuel. The DTT in the system is then quickly consumed, and the time profile for fibers and subunits in fibers resemble the forward process, Figure S37(c, black). On the other hand, the allowed frequency of injection can be large enough that the system uses up the injected amount of fuel in growing fibers before those fibers again collapse prior to the next allowed time of injection.

Amount injected. The amplitude of the oscillations is nearly linearly related to the amount of hydrogen peroxide injected, given the same allowed frequency of injection and threshold fiber count below which to inject hydrogen peroxide, Figure S37(e). This is because a larger amount of fuel is able to sustain fiber growth for a longer period of time. This agrees with observations of varied initial concentrations of hydrogen peroxide.

Fiber threshold. The midpoint of the oscillations depends upon the threshold number of fibers to maintain. The larger the threshold in number of fibers, the higher the axis of oscillations, Figure S37(c). This is because more subunits are required for a larger number of fibers to be sustained.

Efficiency. The amount of hydrogen peroxide injected in the course of this protocol is more than an order of magnitude less than the initial amount of hydrogen peroxide in solution. However, these small injections can sustain fiber growth significantly longer than the synchronous process alone. Measure the efficiency of this protocol, we define two measures of efficiency: the number of fibers present per count of hydrogen peroxide and the count of subunits in fibers per count of hydrogen peroxide. When we look at the profile of these efficiencies in time, they oscillate with significant amplitude at long times—longer times than when the normal synchronous process functions, Figure S37 (a-b). This means that the efficiency of the injection process (as measured by either of these two quantities) is significantly greater than the efficiency of the initial synchronous assembly process.

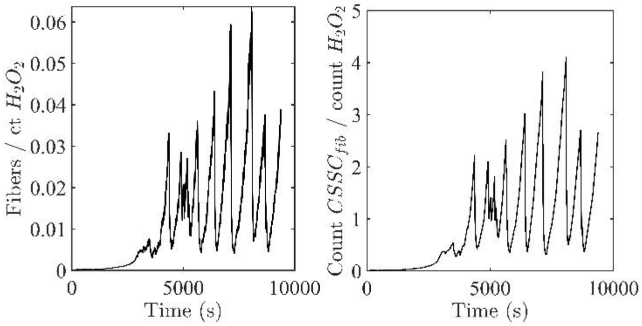

Figure S38. (a) Time profile of number of fibers per count of hydrogen peroxide in simulations reported in $\Delta t = 0.1\ s$ increments with a threshold for injection of 50 fibers, an injection amount of 500 molecules of hydrogen peroxide, and an allowed frequency of injection of 5 s. **(b)** Time profile of count of subunits in fibers per count of hydrogen peroxide in simulations reported in $\Delta t = 0.1\ s$ increments with a threshold for injection of 50 fibers, an injection amount of 500 molecules of hydrogen peroxide, and an allowed frequency of injection of 5 s.

Inject hydrogen peroxide at regular intervals

From the above, we modified the protocol of injection so that rather than injecting with some allowed frequency *when there are sufficiently few fibers*, the hydrogen peroxide is injected at some rate defined by a frequency of injection and an amount to inject (i.e., the threshold condition is removed). In doing this, we found that the frequency of injection does not matter if the equivalent rate is the same (i.e., 100 molecules / 10 s is the same rate as 50 molecules / 5 s), Figure S39(a). A smaller rate of injection in this protocol resulted in a longer period of fiber growth, Figure S38(b). Further, the oscillations in both count of subunits in fibers and fiber counts at late times in the previous protocol are not present using this simplified injection protocol.

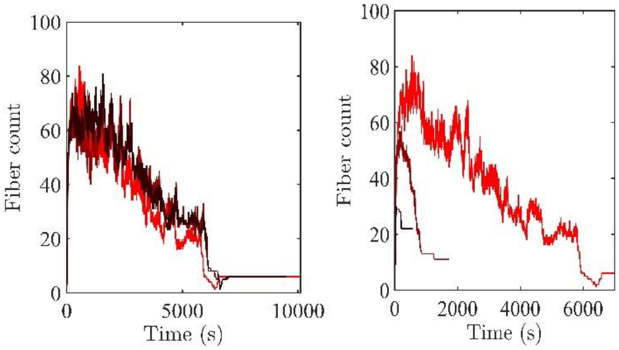

Figure S39. (a) Fiber count over time for equivalent rate of 100 molecules/s injected with injection frequency of 1 s (red), 10 s (dark red), and 60 s (black), reported in $\Delta t = 0.1\ s$ increments. **(b)** Fiber count over time for an injection frequency of 1 s and an injection amount of 100 molecules (red), 1,000 molecules (dark red), and 10,000 molecules (black).

Maintain an amount of hydrogen peroxide in a simulation by injection of hydrogen peroxide

We next attempted to maintain a given amount of hydrogen peroxide fuel in to test the hypothesis that having excess fuel allows for prolonged fiber growth. When we do this, there are oscillations in the fiber growth for a period of time, Figure S40. In this time, DTT^- is also available in solution to scavenge subunits and deactivate them. Once all of the DTT in solution has been deactivated to DTT_{cyc}, fibers grow until there are no more subunits available to add to fibers or create new fibers. There are then small fluctuations in the length of fibers and the number of subunits in those fibers. This is due to Reaction 4, which allows subunits to associate and dissociate from fibers and add to other fibers. However, no fiber collapse then occurs because there is no DTT available to facilitate this. Therefore, fuel alone is not sufficient to perpetuate fiber growth.

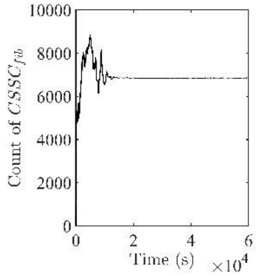

Figure S40. Count of subunits incorporated into fibers in time reported in $\Delta t = 0.1\ s$ time increments looking to maintain 100,000 molecules of hydrogen peroxide in solution by injecting 100 molecules of hydrogen peroxide.

Diffusion limitation of k_6

Using the rate constants optimized for the forward and reverse processes resulted in simulation timescales of the synchronous process that were too short when compared to experimental timescales, suggesting a mechanism in the synchronous process not present in the isolated forward and reverse processes. We hypothesized that the mechanism causing this discrepancy was the speed of molecular diffusion when fibers are present in solution. To model this effect and match the timescale of experiments in the synchronous process, we modified the rate constant or the scavenging of subunits by DTT^- (k_6), decreasing its effective value based on the number of subunits in fibers at each time during the simulation. (We also attempted to account for this behavior using modifications to this rate constant based on the number of fibers in solution and the mean length of fibers in solution, but these did not appreciably change the timescale of the synchronous process.)

In the data reported for the synchronous process, we modified k_6 after each reaction based on count of $CSSC_{fib}$ in the simulation at that time:

$$k_6' = k_6\left(1 - \frac{1}{\left\lfloor\frac{CSH_i}{2}\right\rfloor + 1} \times [fiber\ count]\right)$$

Increasing the fiber count decreases the value of $k_6{}'$. This is normalized by the maximal amount of CSSC that can be in solution, which is given by the initial count of CSH molecules in the simulation integer divided by 2, plus 1 to ensure that the rate constant cannot have a value of zero. This formula ensures that the effective value of the rate constant is within the range $0 < k_6' \leq k_6$.

Using the above formula to modify k_6 after every reaction in the simulation, we again optimized the synchronous process to best match the timescale of experiments. The best matching simulations of the synchronous process to experiments in terms of time that did not sacrifice the matching of timescales for the forward and reverse processes extended the time of simulations of the synchronous process to 4320 s. This is about 1000 s longer than without this diffusion-based modification. However, this is still significantly shorter than the timescale of experiments (~10 hr).

Diffusion-limited bimolecular reaction rate constants

In the following section, we applied a limitation to the speed at which subunits could be scavenged from solution based upon the idea that the fiber content in solution limits the diffusion of species and thereby slowing reactions. This slowed the overall timescale of the synchronous process. However, the timescale resulting from the previous modification was insufficient to match the experimental timescale—the synchronous process still finished too quickly in simulations. Due to this, we add the same limitation on diffusion to all bimolecular reactions in the mechanism: Reactions 2, 3, 6, 7, and 9.

In order to modify these reactions in the same way as previously, we need to apply the same formula to the rate constants k_1, k_3, k_4, k_5, and k_6. Each of these rate constants was modified according to the formula (analogous to the previous):

$$k' = k\left(1 - \frac{1}{\left\lfloor\frac{CSH_i}{2}\right\rfloor + 1} \times [fiber\ count]\right)$$

This imposes an additional limitation on the chemically-driven synchronous system. Each bimolecular reaction in the mechanism is now limited by the amount of fiber content in the system at a given time due to the limitation on diffusion of having solid fibers in the system.

References

1 D. Danino, Y. Talmon and R. Zana, *Colloids and Surfaces A: Physicochemical and Engineering Aspects*, 2000, **169**, 67–73.

2 E. Nativ-Roth, O. Regev and R. Yerushalmi-Rozen, *Chemical Communications*, 2008, **0**, 2037–2039.

3 J. J. De Yoreo, P. U. P. A. Gilbert, N. A. J. M. Sommerdijk, R. L. Penn, S. Whitelam, D. Joester, H. Zhang, J. D. Rimer, A. Navrotsky, J. F. Banfield, A. F. Wallace, F. M. Michel, F. C. Meldrum, H. Cölfen and P. M. Dove, *Science*, 2015, **349**, aaa6760.

4 E. M. Pouget, P. H. H. Bomans, J. A. C. M. Goos, P. M. Frederik, G. de With and N. A. J. M. Sommerdijk, *Science*, 2009, **323**, 1455–1458.

5 S. B. Nicholson, R. A. Bone and J. R. Green, *J. Phys. Chem. B*, 2019, **123**, 4792–4802.

6 R. Erban, J. Chapman and P. Maini, 2007.

7 D. T. Gillespie, *J. Phys. Chem.*, 1977, **81**, 2340–2361.

8 R. Zwanzig, *Nonequilibrium Statistical Mechanics*, Oxford University Press, 2001.
